# Host adaptation and genome evolution of the broad host range fungal rust pathogen, *Austropuccinia psidii*

**DOI:** 10.1093/g3journal/jkaf255

**Published:** 2025-10-28

**Authors:** Zhenyan Luo, Peri A Tobias, Lavi Singh, Chongmei Dong, Alyssa M Martino, Elle Saber, Maria C Quecine, Nelson S Massola, Lilian Amorim, Peng Zhang, Jianbo Li, Smriti Singh, Ziyan Zhang, Ashley Jones, Robert F Park, Benjamin Schwessinger, Richard J Edwards, Thais R Boufleur

**Affiliations:** Research School of Biology, The Australian National University, Canberra, ACT 2601, Australia; School of Life and Environmental Sciences, The University of Sydney, Camperdown, NSW 2006, Australia; Research School of Biology, The Australian National University, Canberra, ACT 2601, Australia; School of Life and Environmental Sciences, Plant Breeding Institute, University of Sydney, Cobbitty, NSW 2570, Australia; School of Life and Environmental Sciences, The University of Sydney, Camperdown, NSW 2006, Australia; Research School of Biology, The Australian National University, Canberra, ACT 2601, Australia; Luiz de Queiroz College of Agriculture, University of São Paulo, Piracicaba, São Paulo 13418-900, Brazil; Luiz de Queiroz College of Agriculture, University of São Paulo, Piracicaba, São Paulo 13418-900, Brazil; Luiz de Queiroz College of Agriculture, University of São Paulo, Piracicaba, São Paulo 13418-900, Brazil; School of Life and Environmental Sciences, The University of Sydney, Camperdown, NSW 2006, Australia; School of Life and Environmental Sciences, The University of Sydney, Camperdown, NSW 2006, Australia; School of Life and Environmental Sciences, The University of Sydney, Camperdown, NSW 2006, Australia; Research School of Biology, The Australian National University, Canberra, ACT 2601, Australia; Research School of Biology, The Australian National University, Canberra, ACT 2601, Australia; School of Life and Environmental Sciences, Plant Breeding Institute, University of Sydney, Cobbitty, NSW 2570, Australia; Research School of Biology, The Australian National University, Canberra, ACT 2601, Australia; School of Life and Environmental Sciences, Plant Breeding Institute, University of Sydney, Cobbitty, NSW 2570, Australia; Research School of Biology, The Australian National University, Canberra, ACT 2601, Australia; Luiz de Queiroz College of Agriculture, University of São Paulo, Piracicaba, São Paulo 13418-900, Brazil

**Keywords:** Pucciniales, mating-type, transposable elements, DNA methylation, transcriptome, phased genome assembly

## Abstract

Rust diseases on plants are caused by fungi in the order Pucciniales. Typically, rust fungi have narrow host specificity however the pandemic biotype of *Austropuccinia psidii* has an unusually broad host range causing disease on over 480 myrtaceous species globally. We assembled and analyzed a fully phased chromosome-level genome for the pandemic *A. psidii* and addressed key outstanding questions of its infection biology. Our research revealed a conserved rust fungal karyotype of 18 haploid chromosomes, in line with fungi for distantly related cereal rusts. We observed chromosomal re-assortment between the 2 nuclei, with one nucleus carrying 19 and the other 17 chromosomes. The synteny of universal single-copy orthologs is mostly maintained with the distantly related rust fungus *Puccinia graminis* f. sp. *tritici*. In contrast, nucleotide composition and methylation profiles of *A. psidii* are distinct compared to rust fungi with smaller genome sizes that have not undergone massive transposable element expansions. Our analysis of MAT loci supports a tetrapolar mating system for *A. psidii* with a novel finding of expanded numbers of pheromone peptide precursors. We show that infection dynamics of *A. psidii* are consistent on 4 different susceptible host species separated by 65 mya of evolution and that transcriptional regulation during infection reveals 2 distinct waves of gene expression in early and late infection, including allele-specific expression of candidate effectors. Together, these findings enhance the understanding of the genome biology and pathology of *A. psidii*, while also providing a valuable resource for future research on this serious rust pathogen.

## Introduction

Rust diseases, caused by obligate biotrophic fungi from the order Pucciniales, have historically devastated economies and contributed to famines worldwide (Oliver 2024). The Pucciniales are one of the largest plant pathogenic fungal orders (Aime and McTaggart 2021), and although they typically display a high degree of host specificity due to coevolution (Aime et al. 2017), species such as *Phakopsora pachyrhizi*, *Phakopsora meibomiae*, and *A. psidii* infect a wide range of hosts within leguminous plants (Ono et al. 1992; Bonde et al. 2008; Slaminko et al. 2008) and the Myrtaceae (Soewarto et al. 2019), respectively.

Originally classified as a monospecific genus, *Austropuccinia* was defined by a single species *A. psidii* (Beenken 2017 ) until 2024 when *Austropuccinia licaneae* (Henn) Ebinghaus & Dianese, *comb. nov.*, was taxonomically proposed as a fungus that infects *Licania* trees from the Chrysobalanaceae family in the Amazons (Ebinghaus et al. 2024). The broad host range of *A. psidii* is not universally applicable to all pathogen isolates and biotypes. Within its native range of South America, most biotypes exhibit various degrees of host specificity, for example, the guava biotype does not sporulate on *Syzygium jambos* (Graça et al. 2013; Stewart et al. 2017; Morales et al. 2024). Outside its native range, the behavior of biotypes might be different. For example, a recent study showed that the South American eucalyptus biotype of *A. psidii* produced infection symptoms on several New Zealand myrtaceous species not found in South America (Soewarto et al. 2025). Further, the pandemic biotype of *A. psidii* has been shown to infect over 480 Myrtaceae globally (Stewart et al. 2017; Carnegie and Pegg 2018) and, as a highly invasive pathogen, has caused dramatic impacts to natural ecosystems notably in Australia, New Zealand, and Hawaii (Berthon et al. 2019; Fensham et al. 2020; Fensham and Radford-Smith 2021).

*A. psidii*, like other rust fungi, exists predominantly in the asexual dikaryotic urediniospore stage of its life cycle, with 2 separate haploid nuclei coexisting in the same cytoplasm. A key goal in eukaryotic genome evolution research is achieving fully phased genome assemblies with telomere-to-telomere (T2T) chromosomes. Rust fungi genome sizes ranges from ∼70 Mb to ∼3 Gb (Tavares et al. 2014; Ramos et al. 2015), and long reads alone are insufficient to assemble and phase dikaryotic genomes completely especially of sizes greater 1 Gb (Tobias et al. 2021). Until very recently, the most effective approach combines PacBio High-Fidelity (HiFi) reads (Wenger et al. 2019) with Oxford Nanopore Technologies (ONT) ultra-long reads and long-range chromatin interaction data such as Hi-C (Illumina) (Li and Durbin 2024). Currently, the few rust fungal genomes that have been fully phased and assembled to near-T2T are primarily host-specific cereal rusts with a karyotype of 18 chromosomes (Duan et al. 2022; Henningsen et al. 2022; Schwessinger et al. 2022; Li et al. 2023; Liang et al. 2023; Sperschneider et al. 2025; Tam et al. 2025). A partially phased genome for the pandemic lineage of *A. psidii* based on PacBio RSII and Sequel technologies revealed over 90% transposable elements (TEs), unusually large telomeres, and a haploid genome size of around 1 Gb. However, scaffolding and phasing of this *A. psidii* genome (v1) (Tobias et al. 2021) were challenging due to early-generation PacBio read error and long-read assembly software. The v1 genome therefore incorporated a considerable number of residual errors, especially around highly repeat-rich regions like centromeres and mating-type (MAT) loci.

The large genome size of *A. psidii* is hypothesized to result from bursts of TEs and their potential silencing via methylation and cytosine deamination which might have led to the comparatively low GC content, at 33.8% (Tobias et al. 2021). Understanding the mechanisms behind genome size expansion in fungal pathogens and how TE activity contributes to this phenomenon are critical questions in fungal biology and evolution (Fouché et al. 2022). TEs are repeated sequences that enable their own proliferation within the genome (Fouché et al. 2022), with roles in chromosome rearrangement and gene expression regulation (Bourgeois and Boissinot 2019). Because uncontrolled proliferation can lead to genome instability (Mita and Boeke 2016), fungi have evolved defense mechanisms that include RNA interference (RNAi), repeat-induced point (RIP) mutation, DNA, or histone methylation (Selker 1990, 2002; Buchon and Vaury 2006; Zhou et al. 2020). How these TE silencing mechanisms contribute to genome evolution in rust fungi including *A. psidii* is currently poorly understood.

TEs also play an important role in MAT loci evolution in rust fungi (Luo et al. 2025). Genes at the MAT loci regulate mating in fungi (Turgeon 1998), which exhibit either bipolar or tetrapolar mating systems (Coelho et al. 2017). In most Basidiomycota, successful mating requires the presence of heterozygous pheromone and receptor (PR) and homeodomain (HD) loci (Hsueh and Heitman 2008). In rust fungi, the PR locus consists of one pheromone gene (*Pra*) and at least one pheromone peptide precursor gene (*mfa*), while the HD locus contains 2 tightly linked HD transcription factor genes (*bW-HD1* and *bE-HD2*) (Coelho et al. 2017; Cuomo et al. 2017). Using an early version of the near-T2T *A. psidii* genome (Edwards et al. 2022), *Pra* and *HD* genes were identified, but no putative *mfa* genes (Ferrarezi et al. 2022). Further analyses revealed that this species most likely has a tetrapolar mating system characterized by a multiallelic HD locus and a biallelic PR locus, facilitating outcrossing on universally susceptible hosts (Ferrarezi et al. 2022; Luo et al. 2025). The MAT loci were poorly resolved in the v1 *A. psidii* genome, meaning that detailed information available on the genome biology and linkage of MAT loci in *A. psidii* was lacking. Recent studies on phased genomes of cereal rust species supported the hypothesis of a tetrapolar mating system in rust fungi by revealing the MAT loci on 2 different chromosomes, multiallelic HD genes and a biallelic PR locus, with the exception of *P. graminis* f. sp. *tritici*, which potentially possesses multiple alleles at the PR locus (Luo et al. 2025). The pandemic lineage of *A. psidii* has an expanding list of host species partly explained by its global spread (Carnegie and Pegg 2018; Soewarto et al. 2019; Boufleur et al. 2023; Ruffner et al. 2024), and the results of several studies suggest that it has different infection dynamics on different hosts (Yong et al. 2019; Beresford et al. 2020; Smith et al. 2020; Martino et al. 2024). While symptoms across multiple host species in controlled inoculations are reported to vary considerably (Morin et al. 2012; Chen et al. 2025; Soewarto et al. 2025), to our knowledge, no study has investigated infection dynamics only within highly susceptible host plants of different species. We investigated this by testing infection dynamics and pustule development within previously determined susceptible individuals from 4 species separated by 65 mya of evolution.

This research confirms the conserved karyotype in rust fungi, identifies unusual chromosome assortment, and addresses 4 fundamental knowledge gaps around *A. psidii* biology that are important to develop long-term pathogen containment. Based on these knowledge gaps combined with new resources for *A. psidii*, we posed the following 6 key questions to investigate: (i) Does *A. psidii* have distinct infection progression in different susceptible host species? (ii) What is the karyotype of *A. psidii*? (iii) What is the conserved gene synteny compared to distantly related rust fungi? (iv) Does DNA methylation play a role in TE silencing? (v) What is the genome biology of MAT loci? (iv) How prevalent is allele-specific expression during the infection process?

## Materials and methods

### Infection assay

Plants from 4 different species belonging to the Myrtaceae family were used in this experiment, including *Leptospermum scoparium*, *Melaleuca alternifolia*, *Melaleuca quinquenervia*, and *S. jambos*. The plants were selected based on confirmed susceptible phenotype from controlled inoculations (Chen et al. 2025; Martino et al. 2024). Plants were cultivated in a peat and perlite mixture (1:5) and grown in a greenhouse at controlled temperatures of 24 °C during the day and 20 °C at night on a 12 h cycle.

*A. psidii* urediniospores of isolate Au3 (Tobias et al. 2021), which belongs to the pandemic biotype, were amplified on *S. jambos* and used for all subsequent infection experiments. Urediniospores from 5-d-old pustules were harvested by shaking, suspended in 0.05% Tween 20, and adjusted to 10^6^ urediniospores mL^−1^. The urediniospore suspension was painted on young leaves of the second internode (both abaxial and adaxial surfaces) for all plants, as described previously (Yong et al. 2019). Plants were incubated for 24 h in a dark sealed box with plentiful water to maintain high humidity, at 23 °C, and subsequently moved to a growth chamber and maintained at 22 ± 2 °C, 60% relative humidity, and a 14 h light photoperiod. Three biological replicates were inoculated for each species, with each biological replicate corresponding to a distinct plant individual.

To compare infection symptom development, multiple selected leaves were manually inspected from one to 12 d post-inoculation (dpi) to register the occurrence of the first visible symptoms, along with recording images of all leaves. Both adaxial and abaxial surfaces of individual leaves were documented at a distance of 1 to 2 cm.

### Cytological study

Fresh urediniospores were suspended in sterilized ddH_2_O containing 0.05% Tween 20 at a concentration of 1 mg/mL and mixed by inverting gently. The suspension was spread into a thin layer on 2% water agar plates. The plates were covered with foil and incubated for 24 h at 18 °C to stimulate germination. Germinated urediniospores were suspended in ddH_2_O and centrifuged at 5,000 rpm for 2 min to remove ddH_2_O. Following treatment with nitrous oxide gas at 1.0 MPa for 1.5 h, the urediniospores were fixed in ice-cold 90% acetic acid for 8 min and then centrifuged at 5,000 rpm for 2 min to remove the acetic acid. The tubes were placed on ice and washed twice with distilled water and then centrifuged at 5,000 rpm for 2 min to remove residual water. The urediniospores were digested in 1.5% Lysing Enzymes from *Trichoderma harzianum* (Cat. No. L1412, Sigma) for 2 h at 28 °C. After digestion, they were washed twice with 70% ethanol and then centrifuged at 5,000 rpm for 2 min to remove the enzymes and ethanol. The urediniospores were squashed using a fine needle in 50 µL of 70% ethanol, centrifuged at 5,000 rpm for 2 min, and dried to remove the remaining ethanol. The urediniospores were vortexed at maximum speed in 50 µL of glacial acetic acid to separate cells from one another. The cell suspension was dropped on microscope slides in a humid box and dried slowly.

Chromosomes were observed with a Zeiss Axio Imager epifluorescence microscope, and images were captured with a Retiga EXi CCD camera (QImaging, Surrey, BC, Canada) operated with Image-Pro Plus version 7.0 software (Media Cybernetics Inc., Bethesda, MD, USA).

### PacBio and ONT long-read DNA sequencing

To improve the scaffolding on the previous assembled *A. psidii* (Au3_v1) genome (Tobias et al. 2021), 16 µg of previously obtained high molecular weight (HMW) DNA was sent to the Australian Genome Research Facility (AGRF) at the University of Queensland, Brisbane, Australia, for PacBio Sequel II HiFi sequencing. For ONT sequencing, HMW DNA of Au3 urediniospores preserved at −80 °C was extracted using a modified cetrimonium bromide (CTAB) extraction protocol (Schwessinger 2016; Martino et al. 2024). The extracted DNA was sent to the Biomolecular Resource Facility at the Australian National University (ANU). The DNA was size selected using a BluePippin (Sage Science) with a cutoff of 40 kb+ for library preparation according to the manufacturer's protocol, with the Ligation Sequencing kit V14 (SQK-LSK114). Sequencing was performed on a PromethION using a flow cell FLO-PRO114M according to the manufacturer's instructions. The pbccs v.6.4.0 was used to generate HiFi reads with tag “—mini-rq = 0.88”. Actc v0.6.0 (https://github.com/PacificBiosciences/actc) was applied to map subreads to HiFi reads, followed by processing with DeepConsensus v.1.2.0 for read corrections (Baid et al. 2023). PromethION fast5 reads were base called to fastq with Guppy v.6.4.6 (https://community.nanoporetech.com) with model dna_r10.4.1_e8.2_400bps_hac@v3.5.2. Sequencing quality was checked with Nanoplot v. 1.41.0 (De Coster and Rademakers 2023). ONT reads were processed with Chopper v.0.5.0 (De Coster and Rademakers 2023) retaining the longest 35× fraction and reads with a quality score of ≥Q7.

### Illumina and ONT RNA sequencing

For Illumina RNA sequencing, *S. jambos* plants with young growing tissues were inoculated with *A. psidii* Au3 isolate as described in 2.1. Six fragments of infected leaves located at the first to third internode were collected at one, 2, 3, 4, 5, and 6 dpi, snap frozen in liquid nitrogen and stored at −80 °C for total RNA extraction. *L. scoparium*, *M*. *alternifolia*, *M. quinquenervia* and *S. luehmannii* were sampled following the similar procedure at 3, 6, and 9 dpi. Whole leaves were collected for these species due to their smaller leaf sizes.

Total RNA was extracted from triplicate samples of plant-infected leaf tissues and ungerminated urediniospores of the Au3 isolate. The samples were homogenized using TissueLyser II (25 Hz for 2 min) and the RNA was extracted with the Spectrum plant total RNA kit (Sigma) as per manufacturer's instructions. DNase treatment was carried out using TURBO DNA-free Kit (Thermo Fisher Scientific). Oligo d(T) 25 magnetic beads (New England Biolabs) were used to purify the RNA as per manufacturer instructions. The RNA quantity was assessed using a Qubit RNA kit on a Qubit Fluorometer (Thermo Fisher Scientific). The quality of RNA was determined using a NanoDrop (Molecular Devices), and the integrity was checked by Gel electrophoresis (1% agarose gel). Purified RNA was sent to Azenta Life Sciences for RNA sequencing with strand-specific polyA mRNA selection using 150 bp paired-end Illumina NovaSeq and a sequencing depth of 20 million reads.

For ONT direct RNA sequencing, 3 mg spores per 1 mL Novec 7100 engineered fluid (3MTM) was sprayed onto plants of *S. jambos* with more than 6 juvenile, pink leaves. Plants were then incubated as described in the infection assay section. At 3 and 6 dpi, 2 to 3 g per infected leaves were collected, snap frozen in liquid nitrogen and stored at −80 °C. Total RNA was extracted using an adapted CTAB protocol (Chang et al. 1993; Inglis et al. 2018), resuspended in 10 mM Tris-HCl pH 7.5, and stored at −80 °C. A NanoDrop spectrophotometer and Qubit RNA Broad Range Assay Kit (both by Thermo Fisher Scientific) were used for RNA quantification and quality control, and the integrity was checked by Gel electrophoresis (1% agarose gel). Dynabeads Oligo (dT)25 (Invitrogen) was used to purify 2 µg poly(A) tailed RNA (mostly mRNA) from total RNA following the manufacturer's instructions. Quality control was performed as described above. Approximately 500 ng of poly(A) transcripts were used as input for the direct RNA sequencing library. RNA reverse transcription was done with Induro Reverse Transcriptase following the Induro Reverse Transcriptase Protocol (NEB M0681). The remaining library preparation and ONT Direct RNA sequencing kit (SQK-RNA002) were performed using FLO-MIN106D MinION flow cell as per ONT manufacturer's instructions. Approximately 2 million reads per library were obtained. The raw RNA sequencing signals captured in ONT fast5 files were base called with Guppy v.6.4.2 (ONT) using the high accuracy config model (rna_r9.4.1_70bps).

### Genome assembly and scaffolding with Hi-C data

The data were assembled using 18.5× of HiFi and 39.5× of Nanopore read depths per haplotype, supplemented with 80 Gb of raw Hi-C data (Edwards et al. 2022), utilizing Hifiasm software (v.0.19.5-r592) (Cheng et al. 2021, 2022) using default settings. HiFi and ONT reads were mapped individually to the draft assembly with MiniMap2 v2.24 (Li 2021). The per-contig coverage was estimated with BBMap v.38.90 pileup.sh script (https://github.com/BioInfoTools/BBMap/tree/master). Contigs with low coverage were extracted with SAMtools v.1.1 (Danecek et al. 2021) and HiFi and ONT reads that mapped to these contigs were identified and removed from the fastq files with BBmap v.38.93 (https://github.com/BioInfoTools/BBMap/tree/master). A new assembly was run with the filtered reads as described above.

The 2-phased genome outputs were merged, and scaffolding was performed using Hi-C data, following the Aiden Lab pipelines (https://github.com/aidenlab). The merged file was run through the Juicer pipeline (v.1.6) with default parameters (Durand et al. 2016a, 2016b). The final output from Juicer was used with the 3D-DNA pipeline (v.180922) with the following parameters “-m diploid –build-gapped-map –sort-output” (Durand et al. 2016a, 2016). The output of 3D-DNA was manually curated within the Juicebox visualization software (v.1.11.8 for Mac) (Durand et al. 2016a, 2016), and the revised assembly file was resubmitted to the 3D-DNA post review pipeline with the following parameters “—build-gapped-map –sort-output” for final assembly and FASTA files. For post-scaffolding contamination removal and clean-up, unplaced contigs were BLASTed against the RefSeq database of NCBI for contaminant identification.

Functional completeness of each haploid genome and from the dikaryon was estimated using BUSCO v.5.4.0 (Simão et al. 2015) with the basidiomycota_odb10 lineage database. The most complete haplotype was named hapA and the chromosomes were arranged by size and each starting with the short chromosome arm. HapB was mapped to hapA to identify and name homologous chromosomes and organize unscaffolded contigs by size with PAFScaff v.0.6.3 (Field et al. 2020). Terminal telomeric repeats were predicted using Telociraptor v0.8.0 (https://github.com/slimsuite/telociraptor). The final assembly was designated as the v3 assembly (hapA, hapB, and mt).

### Repeat and gene annotation

RepeatMasker v.4.0.9 was applied to reannotate the genome with a de novo constructed TE database from Au3_v1 (Tobias et al. 2021). The Wicker classification system (Field et al. 2020) was applied to classify TEs into the superfamily level. For TE families that could not be classified by REPET v.3, new classifications were assigned based on TE sequences showing at least 70% identity to the consensus sequences of these TE families. For calculating TE density, all insertions were included in the analysis. To avoid bias in analyzing the relationship between TEs and methylation, only insertions longer than 1 kbp were included.

The haploid genomes were soft-masked and functionally annotated using the Funannotate v.1.8.15 pipeline (https://zenodo.org/record/2604804). The prediction includes “train”, “predict”, and “update”. The transcriptome generated by ONT and Illumina sequencing was used in the “train” step, and “–jaccard_clip” was added for better fungal annotation. In the “predict” step, UniProt database was added as protein hints for the first round, and the weight of each gene prediction tools was set as the default. In the “update” step, the parameter of “–jaccard_clip” was added. After the first round of annotation, protein files generated in opposite haplotypes were added as protein hints in the “predict” step for the second round.

Given the repeat-rich nature of the *A. psidii* genome, the predicted genes might be related to TEs despite soft-masking, which could influence subsequent analysis. To address this, Orthofinder v.2.5.5 (Emms and Kelly 2015) was employed to identify orthogroups between *A. psidii*, *Pst 104E*, and *Pt 76*. Most orthogroups have less than eight gene members and less than 20 orthogroups of *Pst 104E* and *Pt 76* have more than 20 gene members, so orthogroups containing more than 20 gene members in *A. psidii* were classified as repeat-related genes and were removed from further analysis.

The predicted genes were functionally annotated using the “funannotate annotate” command in the pipeline and described as follows. Protein-coding gene models were used to pull annotations from Pfam, dbCAN (CAZyme), and Gene Ontology (GO) databases. GO and Pfam enrichment analysis were implemented using “enricher” function in clusterProfiler v.4.12.6 R package (Wu et al. 2021), which fits a hypergeometric test to identify over-represented terms (Benjamini–Hochberg false discovery rate [FDR] of *P* < 0.05). To predict the secretome of the *A. psidii* isolate Au3, proteins containing a signal peptide cleavage site were identified with SignalP v.6.0 (Teufel et al. 2022). Subsequently, proteins with transmembrane (TM) domains and glycosylphosphatidylinositol (GPI) anchoring were identified with DeepTMHMM v.1.0.39 (Hallgren et al. 2022) and NetGPI v.1.1 (Gíslason et al. 2021). Proteins with a signal peptide cleavage site and absence of TM domain and GPI-anchors were predicted to be part of the *A. psidii* secretome. The subcellular localization of the secretome was predicted using WoLF PSORT (Horton et al. 2007). Finally, blastp v.2.12.0+ (Altschul et al. 1990; Johnson et al. 2008) was employed to detect secreted proteins that were either shared among haplotypes or hemizygous, applying criteria of at least 90% identity and 50% query coverage. Blast results were checked manually and the best hit in both haplotypes was used as a criterion to identify the alleles. Secreted proteins that were differentially expressed in planta when compared to ungerminated urediniospores were classified as the candidate effectors (CEs) of *A. psidii*. The pattern of expression of the identified alleles (shared) and specific (hemizygous) CEs was plotted with pheatmap R package v.1.0.12 (Kolde 2019).

### Chromosome synteny analysis

Chromosome synteny analysis was performed using single-copy “Complete” genes from the BUSCO genome completeness runs. For this analysis, 1 copy of Chr14 was placed in hapB. Synteny blocks between pairs of assemblies were determined by collinear runs of matching BUSCO gene identifications. For each assembly pair, BUSCO genes rated as “Complete” in both genomes were ordered and oriented along each chromosome. Synteny blocks were then established as sets of BUSCO genes that were collinear and uninterrupted (i.e. sharing the same order and strand) in both genomes, starting at the beginning of the first BUSCO gene and extending to the end of the last BUSCO gene in the block. We note that local rearrangements and breakdowns of synteny between BUSCO genes will not be identified and may be falsely marked as syntenic within a synteny block.

Chromosome synteny was then visualized by arranging chromosomes for each assembly in rows and plotting the synteny blocks between adjacent assemblies. In each case, the chromosome order and orientation were set for one “focus” assembly, and the remaining assemblies arranged to maximize the clarity of the synteny plot, propagating out from the focal genome. For each chromosome, the best hit in its adjacent assembly was established as that with the maximum total length of shared synteny blocks, and an anchor point established as the mean position along the best hit chromosome of those synteny blocks. Where the majority of synteny was on the opposite strand, the chromosome was reversed and given an “R” suffix in the plot. Chromosomes were then ordered according to their best hits and, within best hits, the anchor points. Synteny blocks sharing the same orientation were plotted blue, while inversions are plotted in red. Code for establishing and visualizing chromosome synteny based on shared BUSCO genes has been publicly released as a new tool, ChromSyn, under a GNU General Public License v.3.0 and is available on GitHub (https://github.com/slimsuite/chromsyn). Synteny of the hapA and hapB v3 *A. psidii* genomes was analyzed with ChromSyn v0.9.3, and further comparisons were also made against the fully phased, chromosome-level wheat leaf rust pathogen genome, *Puccinia graminis* f. sp. *tritici* (*Pgt 21-0*) (Inglis et al. 2018). In addition, Seaborn v0.13.2 (Waskom 2021) was used to generate heatmaps that show the number of BUSCOs that are shared between chromosomes of *A. psidii* and species within or outside the order Pucciniales, to further investigate the conservation of synteny. Query chromosomes were reordered based on the best match to Au3 subject chromosomes. Species and assemblies used for comparison are *Melampsora lini* CH5 (Sperschneider et al. 2025), *P. graminis* f. sp. *tritici* 21-0 (GCA_008522505.1) (Li et al. 2019), *Ustilago bromivora* UB2112 (GCA_900080155.1) (Rabe et al. 2016), and *Sporisorium panici-leucophaei* SPL10A (GCA_014826065.1) (Crestana et al. 2021). The Dnadiff module from Mummer v4.0.0rc1 was used to calculate the average nucleotide identity between haplotypes with 1 of chromosome 14 was placed in haplotype B for better comparison (Marçais et al. 2018).

For the nucleotide synteny analysis, chromosomes of hapA and hapB v3 were aligned with Minimap2 v.2.28 (with options: –cs -cx asm20 –secondary = yes) (Li 2021). Matches with at least 80% identity and 1 kbp in length were used for generating a dotplot with Matplotlib v.3.9.1 (Hunter 2007).

To perform gene based synteny analysis, orthologs between haplotypes were identified with blastp v.2.15.0 (Altschul et al. 1990; Johnson et al. 2008). Matches were filtered to ensure a minimum of 50% sequence coverage for both query and reference sequences, along with at least 70% identity. TE density and 5mCpG/CpG ratio were calculated using non-overlapping 10 kbp windows, with GenomicRanges v.1.54.1 (Lawrence et al. 2013). The results were visualized with karyoploteR v.3.17 (Gel and Serra 2017).

### Dinucleotide and DNA methylation analysis

For calculating observation/expectation (O/E) ratio of dinucleotides, the following formula was used to calculate the ratio for each chromosome separately (Upadhyay et al. 2014):


$$(O/E)XpY=(O/E)Y^{\prime }pX^{\prime }=2G\times \frac{\;f(XpY)f(Y^{\prime }pX^{\prime })}{\left(\;f(X)+f(Y))(\;f(X^{\prime })+f(Y^{\prime })\right)}$$

where *f* represents frequency, XpY represents the dinucleotide in one strand, Y′pX′ represents the complementary dinucleotide in the opposite strand, and *G* represents the length of the chromosome. HiFi reads with kinetics were generated from raw subreads by pbccs (v.6.4.0, https://github.com/PacificBiosciences/ccs) with the “—hifi-kinetics” tag. Jasmine v.2.0.0 was applied to predict 5mC of each CpG site from PacBio HiFi reads, and pbmm (v.2 1.14.99, https://github.com/PacificBiosciences/pbmm2.git) was then used for aligning HiFi reads to the genome. Pb-CpG-tools (v.2.3.2, https://github.com/PacificBiosciences/pb-CpG-tools.git) was used for generating site methylation probabilities from mapped HiFi reads; only sites that have at least a 50-modification score were regarded as methylated fractions.

The total number of methylated sites within non-overlapping 1 kb windows across the genome was calculated as follows:


$$\begin{array}{rl} \mathrm{Coverage}=\; & \frac{\left[\mathrm{Total}\;\mathrm{number}\;\mathrm{of}\;\mathrm{methylated}\;\mathrm{CpG}\;\mathrm{in}\;1\;\mathrm{kb}\;\mathrm{window}\right]}{\left[\mathrm{Total}\;\mathrm{number}\;\mathrm{of}\;\mathrm{CpG}\;\mathrm{in}\;1\;\mathrm{kb}\;\mathrm{window}\right]}\\ & \times 100\;\text{\%} \end{array}$$

For confirming the 5mC methylated fractions, 5mC base calling and mapping were also performed using Dorado (v0.7.3+, https://github.com/nanoporetech/dorado) with the sup DNA model v.4.1.0.

Statistical differences between hap A and hap B were analyzed using Student's *t*-test by applying the SciPy module ttest_ind (Virtanen et al. 2020) (84). Correlations between TE identity and GC content, as well as between TE identity and counts of CpG fractions per kbp, were analyzed using Pearson's correlation coefficient, applying the SciPy modules pearson (Virtanen et al. 2020).

For examining whether transposons were hypermethylated, a permutation test was performed to compare random occurrences of 5mCpG fractions and observed 5mCpG fractions of TEs, coding sequences (CDS), and untranslated regions (UTRs). Random shuffling of 5mCpG fractions across CpG dinucleotides was performed using bedtools shuffle v.2.31.0 (Quinlan and Hall 2010). The shuffling was constrained to occur only at CpG sites using the following parameters: -chrom (restrict shuffling to the same chromosome), -noOverlapping (prevent overlapping features), and -incl (specify CpG site locations). Random seeds were generated using the random module of Python v.3.12, and the shuffling process was repeated for 1,000 iterations. Percentage of 5mCpG fractions occurred in TEs, CDS and UTRs were calculated with pybedtools v.0.10.0 (Dale et al. 2011), the 2-sided *P*-value was calculated by comparing simulation and observation, visualized with Seaborn v.0.13.2 (Waskom 2021).

### Centromere analysis

Contact maps visualized with Juicebox software (v.2.17.00) (Durand et al. 2016a, 2016b) were used to estimate the position of centromeres. Statistical differences of TE coverage and 5mCpG/CpG ratio between centromeric and non-centromeric regions were performed using paired sample *t*-test by applying the SciPy module ttest_rel (Virtanen et al. 2020).

### MAT analysis

To identify *MAT* genes, the *bW-HD1*/*bE-HD2* and *PRA* genes of *Pt BBBD* isolate (Cuomo et al. 2017) were used as queries in a blastp v.2.15.0 (Altschul et al. 1990; Johnson et al. 2008) search. Following the confirmation of the PRA genes position, all open reading frames (ORFs) ranging from 90 to 300 bp that contained “CAAX” motifs were considered potential pheromone peptide candidates. Tandem repeat sequences were manually assessed for each candidate. Sequences less than 210 bp encoding at least 2 tandem repeats were classified as mfa1. Sequences less than 120 bp with “CAAX-Stop” motifs but lacking tandem repeats were classified as MFA2. Sequences greater than 210 base pairs containing tandem repeats like those in MFA1 were categorized as MFA3, based on observations in *P. graminis* f. sp. *tritici*.

A specific pattern “[Q/E]WGNGSH[X]C” (where X can be any amino acid) was frequently found in the candidate mfa1/3 sequences, consistent with reported mfa1/3 in cereal rust fungi (Cuomo et al. 2017; Luo et al. 2025). This pattern was used for further filtering candidates. To predict mfa2, the “CAAX-Stop” motif was employed to capture ORFs ranging from 30 to 40 amino acids. CDS sequences of the identified mfa candidates were searched with blastn v.2.15.0 (Altschul et al. 1990; Johnson et al. 2008) to identify orthologs, with all hits filtered as described above. The amino acids of the candidate mfas were aligned with ClustalW v.2.1 (Sievers and Higgins 2018), and sequence logo graphs were generated using PlotnineSeqSuit (Cao et al. 2023).

To investigate the structure of the MAT locus, the same approach used by Luo et al. (2025) for detecting genetic degeneration was employed. Proteinortho v.6.0.22 (Lechner et al. 2011) was used to identify one-to-one orthologous gene pairs with on sister chromosomes, applying the -synteny and -singles tags. For each gene pair, CDS-based protein alignments were generated with Muscle v.3.8.1551 (Edgar 2004) with default settings. Subsequently, PAML v.4.9 (Yang 2007) was used to calculate synonymous divergence values from each alignment. TE density, 5mCpG ratio, and gene density were calculated with GenomicRanges v.1.54.1 (Lawrence et al. 2013) in 10 kb sliding windows without overlapping. Results were visualized with karyoploteR v.3.17 (Gel and Serra 2017).

### Allele-specific expression analysis

Raw reads obtained in with Illumina sequencing were inspected using FastQC v.0.12.1 (https://www.bioinformatics.babraham.ac.uk/projects/fastqc/). Fastp v.0.23.4 was used for adapter trimming (Chen et al. 2018). To allow discrimination between haplotypes and avoid ambiguous mappings, we generated a reference transcriptome that combined hapA and hapB predicted proteomes, while maintaining unique identifiers for each haplotype-specific gene copy. Trimmed reads were mapped to this merged reference using HISAT2 v.2.21 (Kim et al. 2019) with parameter “–no-unal” so that only reads that mapped to the *A. psidii* transcriptome were retained for further analysis. Salmon v.0.13.1 (Patro et al. 2017) was used to quantify *A. psidii* transcripts from the reads aligned to the transcriptome. Transcript level counts were imported into R v.4.4.1 (R Core Team 2024) and summarized to gene level counts using the tximport package v.1.32.0 (Soneson et al. 2015).

Allele-specific differential gene expression analysis was carried out to identify which *A. psidii* genes were significantly upregulated in planta compared to urediniospores. The edgeR v.4.2.2 package in R (Robinson et al. 2010) was used as it allows comparisons between multiple treatments in a single analysis. Genes that were lowly expressed were filtered out using “filterbyExpr” function in R. The remaining gene counts were normalized with Trimmed Mean of Means (TMM) method using “calcNormFactors” function in edgeR. The quality assessment and expression profiles of the samples were visualized using principal component analysis (PCA) and Pearson correlation. This was achieved through the “plotMDS” function from the Limma package v.3.60.6 (Ritchie et al. 2015), “cor” function, and pheatmap package v.1.0.12 (Kolde 2019) in R. To identify significantly upregulated genes *in planta* relative to urediniospores, pairwise comparisons between in planta time points and ungerminated urediniospores were made (2 dpi vs urediniospores, 3 dpi vs urediniospores, 4 dpi vs urediniospores, 5 dpi vs urediniospores, 6 dpi vs urediniospores). Genes with a Benjamini–Hochberg FDR of *P* < 0.05 and log-fold change >2 or <−2 were considered biologically significant.

## Results

### *A. psidii* displays uniform infection dynamics on host species separated by 65 million years of evolution

**Fig. 1.**
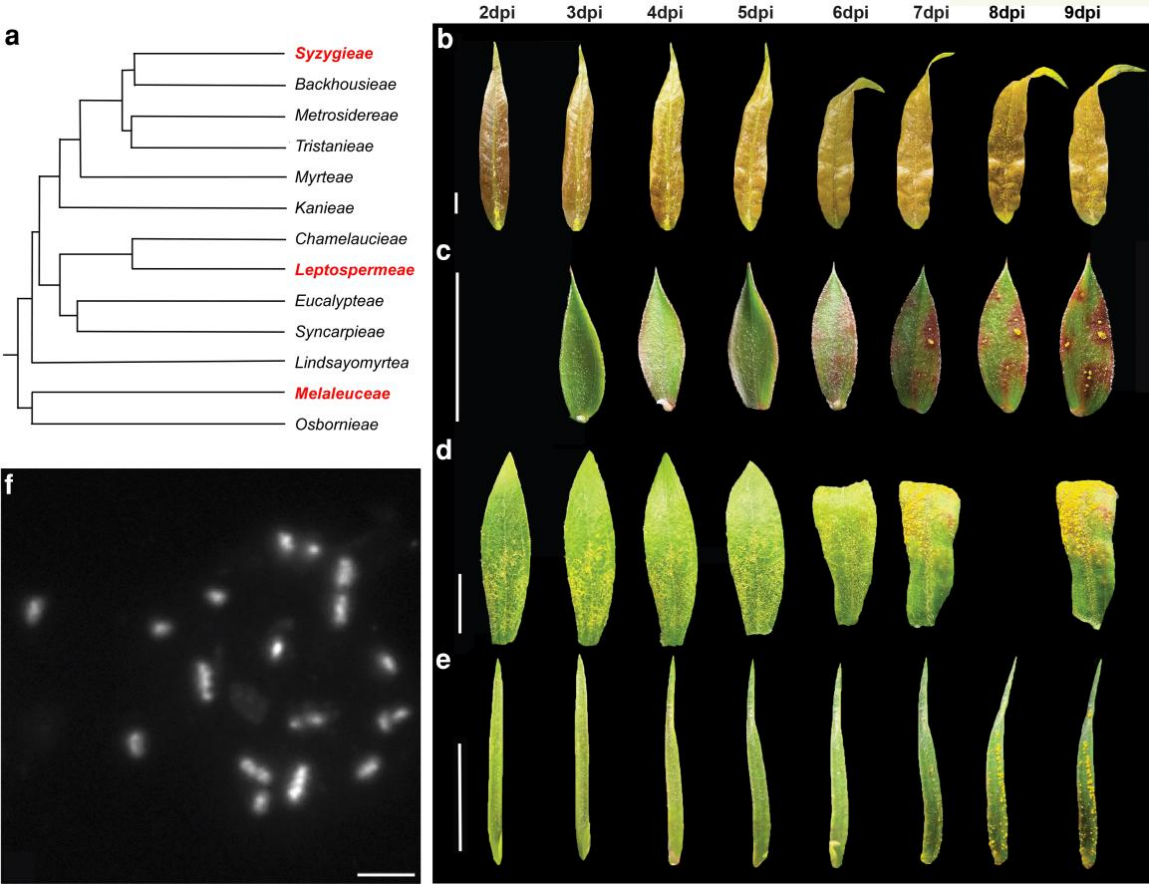
The pandemic biotype of *A. psidii* displays uniform infection dynamics across distantly related plant species. a) Topology of genera within the Myrtaceae family. Genera that have species used in the infection assay are highlighted in red. The figure was adapted from Biffin et al. (2010) and Grattapaglia et al. (2012). b to e) Infection symptoms developed between 2/3 and 9 dpi for b) *S. jambos*, c) *L. scoparium*, d) *M. quinquenervia*, and e) *M. alternifolia*. Scale bars represent 1 cm. f) Cytological analysis of *A. psidii* chromosomes. Chromosomes are counterstained with 4′,6-diamidino-2-phenylindole. They form a bimodal karyotype, with large chromosomes featuring clear primary constriction and 2 chromosome arms, and small chromosomes lacking visible primary constrictions. Scale bar: 2 μm.

To understand the infection dynamics of the pandemic biotype of *A. psidii* on susceptible host species across the Myrtaceae family, we selected 4 species, *S. jambos*, *M. quinquenervia*, *M. alternifolia*, and *L. scoparium*, that are widely distributed across the phylogeny and separated by ∼65 million years of evolution (Fig. 1a) (Berger et al. 2016). Whole-plant infection assays revealed very similar symptom development across all 4 species when incubated under the same conditions (Fig. 1b to e). We did not observe any macroscopic myrtle rust disease symptoms until 6 dpi, when the first lesions appeared, characterized by yellow or reddish spots and leaf curling. By 7 dpi, the initial sporulating pustules emerged, with their numbers increasing steadily until the end of the experiment at nine dpi (Fig. 1b to e; Supplementary Figs. 1 and 2).

### Cytology and near-T2T genome analyses reveal karyotype conservation and inter nuclear chromosome transfer in *A. psidii*

To characterize chromosome size distribution and number in *A. psidii*, we conducted a cytological study. This revealed that *A. psidii* has chromosomes belonging to 2 distinct size classes. In a haploid mitotic metaphase cell, chromosomes were organized into a bimodal karyotype comprising twelve larger and 6 smaller chromosomes (Fig. 1f). The twelve large chromosomes displayed clear primary constrictions and 2 chromosome arms, while the 6 smaller chromosomes lacked visible primary constrictions and were considerably shorter (Supplementary Fig. 3).

We followed up on these cytological karyotype estimations by generating the first near-T2T genome for *A. psidii*, because the previously published *A. psidii* genome assembly was fragmented, with over 66 scaffolds per haplotype and only 3 complete chromosomes (Table 1) (Tobias et al. 2021). This limited the genome biology insights obtained from this partial phased assembly, which contains significant residual assembly errors. To obtain a fully phased near-T2T assembly of the *A. psidii* pandemic biotype, we combined HiFi and ONT long reads, with Hi-C data, followed by additional Hi-C scaffolding (Supplementary Table 2). This approach yielded 2 haploid assemblies of approximately 1 Gbp each, which we named hapA and hapB (Table 1).

**Table 1.** T2T genome assembly statistics of *Austropuccinia psidii* v3.

Genome	# of scaffold (contig)	# of Chr	Total length (bp)	Max length (bp)	N50 (bp)	L50	GC (%)	BUSCO (%)^b^
hap1_v1	66 (3,817)	3	1,018,398,822	124,273,364	56,243,252	7	33.8	C: 88.1 [S: 78.6; D: 9.5]
hapA_v3^a^	…	19	1,073,490,195	85,591,751	60,211,033	8	33.84	C: 91.2 [S: 90.3; D:0.9]
hap2_v1	67 (8,637)	-	934,744,333	89,073,602	52,409,407	7	34.26	C: 70.3 [S: 67.0; D: 3.3]
hapB_v3^a^	…	17	982,558,553	84,687,478	60,506,416	7	33.86	C: 90.9 [S: 90.0; D: 0.9]
Dikarion_v3	(270)	36	2,079,842,500	85,591,751	60,211,033	15	33..86	C: 92.0 [S: 2.8; D: 89.2]

Basic genome statistics comparing the v1 *A. psidii* genome (Tobias et al. 2021), against the current genome assemblies (v3).

^a^Complete BUSCOs (C); complete and single-copy BUSCOs (S); complete and duplicated BUSCOs (D).

^b^To compute BUSCO value, 1 chr14 is distributed into hapB.

**Fig. 2.**
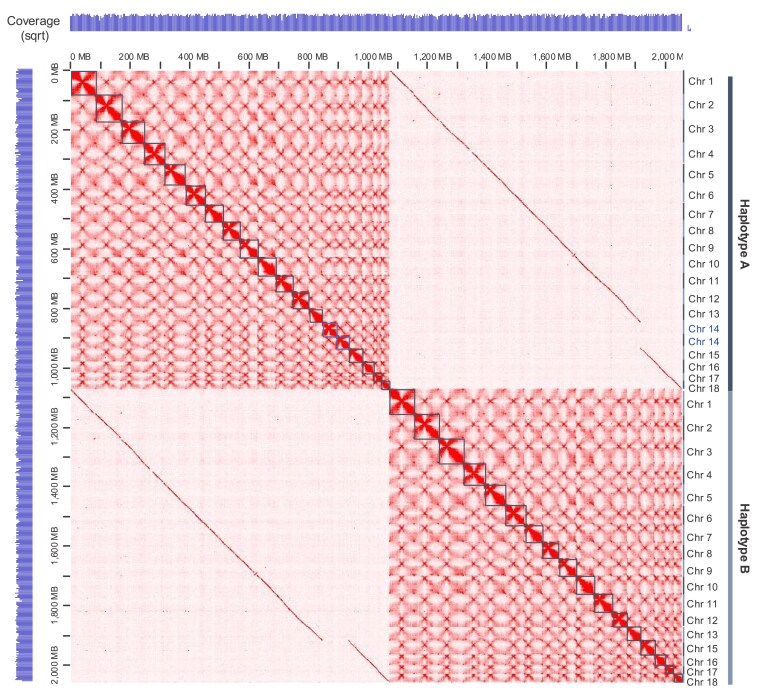
3D genome confirmation capture reveals inter nuclear chromosome transfer in *A. psidii* between its 2 haploid nuclei. 3D genome interaction heatmap of the 36 chromosomes of the *A. psidii* v3 genome visualized in Juicebox. Hi-C coverage (srqt) indicated by pale blue bars, left and top, transformed by taking the square root. Black squares indicate the predicted 19 chromosomes in order of size in haplotype A and 17 chromosomes in haplotype B.

The assembled genome indicates a haploid karyotype of 18 chromosomes of varying sizes, ranging from ∼86 to ∼28 Mb per chromosome (Supplementary Table 3). Surprisingly, Hi-C contact maps (Fig. 2) revealed that hapA contained 19 and hapB 17 chromosome-sized scaffolds, suggesting that chromosome chr14B relocated to the hapA nucleus and is now stably inherited via urediniospores through generations. We ordered and numbered scaffolds by size; hence, scaffold one is the largest of ∼86 Mb and scaffold 18 is the smallest of ∼28 Mb. Fourteen hapA and 15 hapB scaffolds are T2T, indicating likely full-length chromosomes (Table 1 and Fig. 2). The other 5 scaffolds in hapA and 2 scaffolds in hapB have a telomere at 1 end only. Two-thirds (24/36) of the annotated telomeres range between 9 and 12 kb (Supplementary Table 3). The overall BUSCO gene completeness of 92% is a slight improvement over 91.2% for v1. The total genome size for v3 is comparable to v1 (Table 1).

### Gene synteny of conserved genes (BUSCOs) is mostly maintained when compared to distantly related rust fungus *P. graminis* f. sp. *tritici* despite significant expanded genome size due to TEs

First, we confirmed the TE expansion reported for the *A. psidii* v1 genome assembly (Tobias et al. 2021). Approximately 89.14% of the near-T2T genome assembly of *A. psidii* is comprised of repetitive elements. Among these, Class I TEs: LTR, *Ty3* has the highest proportion across all chromosomes, followed by Class II: TIR: CACTA (Supplementary Fig. 4). Then, we tested the gene synteny between the 2 haplotypes of *A. psidii*. Overall, a conserved syntenic relationship was observed across most chromosomes (Supplementary Figs. 5 and 6), except for a large chromosomal inversion on chr7. Additionally, chr14 showed substantial structural variation, marked by multiple translocation events and gene duplications (Supplementary Figs. 5 and 6). To compare the sequence divergence between haplotypes, we computed the average identity and average base identity of pairwise sister chromosomes. The average identity between sister chromosomes ranged from 94.54 to 95.45%, with chr11 having the least and chr1 having the highest average identity (Supplementary Table 4). Chr5 had the lowest average base identity, with only 88.32% for haplotype A and 92.13% for haplotype B. The average base identity of chr14 was 94.02 and 96.02%; both showed no significant differences from the average base identity across all chromosomes.

**Fig. 3.**
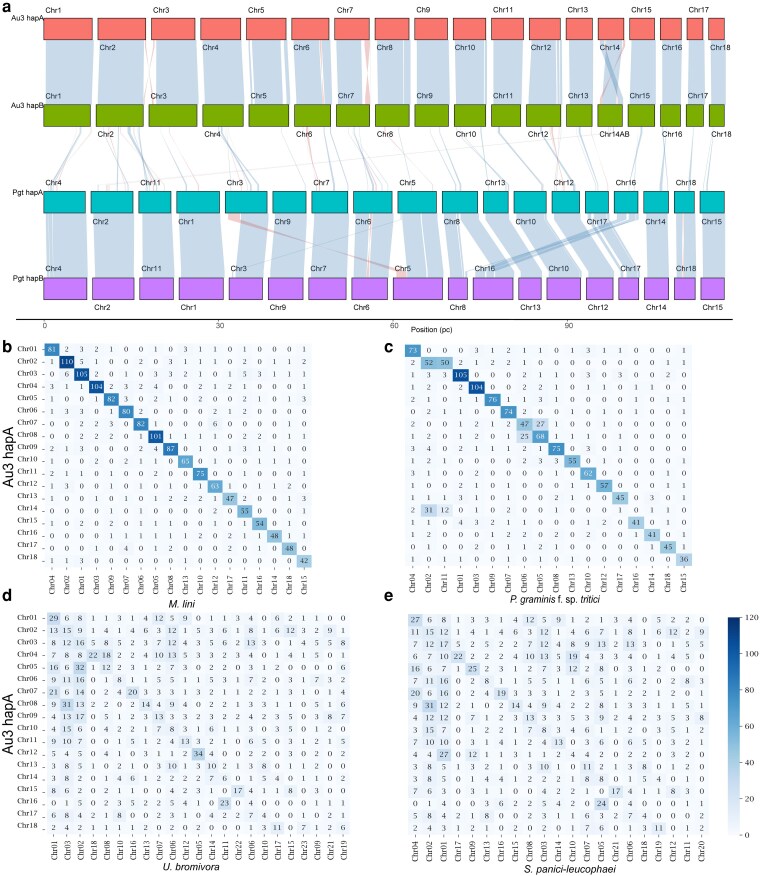
BUSCO gene synteny is largely conserved between *A. psidii* and 2 representative rust fungi. a) The figures show ChromSyn BUSCO-derived synteny plots. Colored blocks represent assembly scaffolds over 1 Mbp for each genome. Synteny blocks of collinear “Complete” BUSCO genes link scaffolds from adjacent assemblies: blue, same strand; red, inverse strand. Filled circles mark telomere predictions from Diploidocus (black) and TIDK (blue). Assembly gaps are marked as dark red +/− signs. Yellow triangles denote duplicated BUSCOs. Genes on the forward strand are marked above the centerline, with reverse strand genes below. Chromosomes are scaled by their size relative to total genome size of each assembly (scale = pc), which normalizes for differences in chromosome size and allows for easier comparison between genomes with significant size differences. Pairs of phased haploid genomes for *A. psidii* v3 (Au3 hapA and Au3 hapB) and *P. graminis* f. sp. *tritici* 21-0 (Pgt hapA and Pgt hapB) are shown (Li et al. 2019). b to e) Heatmaps show counts of shared BUSCOs per chromosome pair between b) *A. psidii* and *M. lini* (Sperschneider et al. 2025), c) *A. psidii* and *P. graminis* f. sp. *tritici* (Li et al. 2019), d) *A. psidii* and *U. bromivora* (Rabe et al. 2016), and e) *A. psidii* and *S. panici-leucophaei* (Crestana et al. 2021).

Since *A. psidii* and other known rust fungi share an identical karyotype, we investigated the conservation of the synteny of BUSCO genes. The synteny of BUSCO genes was compared between the 2 *A. psidii* haplotypes and to the chromosome assembly of *P. graminis* f. sp. *tritici* 21-0 (*Pgt* 21-0) (Li et al. 2019). For this analysis, we developed ChromSync, a tool designed for the rapid identification of syntenic blocks based on BUSCO gene order. The synteny of BUSCO genes is largely conserved between the 2 *A. psidii* haplotypes with hardly any structural translocation of BUSCO genes among chromosomes of the 2 haplotypes. Similarly, the overall synteny of BUSCO genes was mostly conserved between *A. psidii* and *P. graminis* f. sp. *tritici* 21-0 chromosomes except for multiple rearrangements involving chr14 and chr2 of *A. psidii* and chr2 and chr11 of *P. graminis* f. sp. *tritici* 21-0 (Fig. 3a). We analyzed the chromosomal location of BUSCO genes that are shared between *A. psidii* and 4 other fungal species, including 2 within and 2 outside the order Pucciniales, to further investigate the conservation of synteny. The heatmaps of *A. psidii* and *M. lini* or *P*. *graminis* f. sp. *tritici* indicate that most shared BUSCO genes between these species are located on 1:1 chromosome pairs (Fig. 3, b and c). In contrast, the results between *A. psidii* and *U. bromivora* or *S. panici-leucophaei* show less conservation in the number of shared BUSCO genes per chromosome (Fig. 3, d and e).

### Detailed analysis of the *A. psidii* near-T2T genome reveals specific DNA methylation patterns, CpG depletion via historic 5mC deamination, and centromeres

Using the new near-T2T genome assembly, we extended the genome biology analyses of *A. psidii*, focusing on aspects that previously were intractable including CpG DNA methylation, centromeres, and detailed analyses of the MAT loci. Consistent with previous studies, younger TEs (with >90% family-level sequence identity) exhibit higher GC content and more available CpG methylation sites (Supplementary Fig. 7). Both measures were also positively correlated with TE family age when measured as average percentage identity of individual TE insertions relative to the TE family's consensus sequence (family-level sequence identity) (Pearson's *r* = 0.18 and *r* = 0.12 respectively, both *P* < 1 × 10^−16^).

**Fig. 4.**
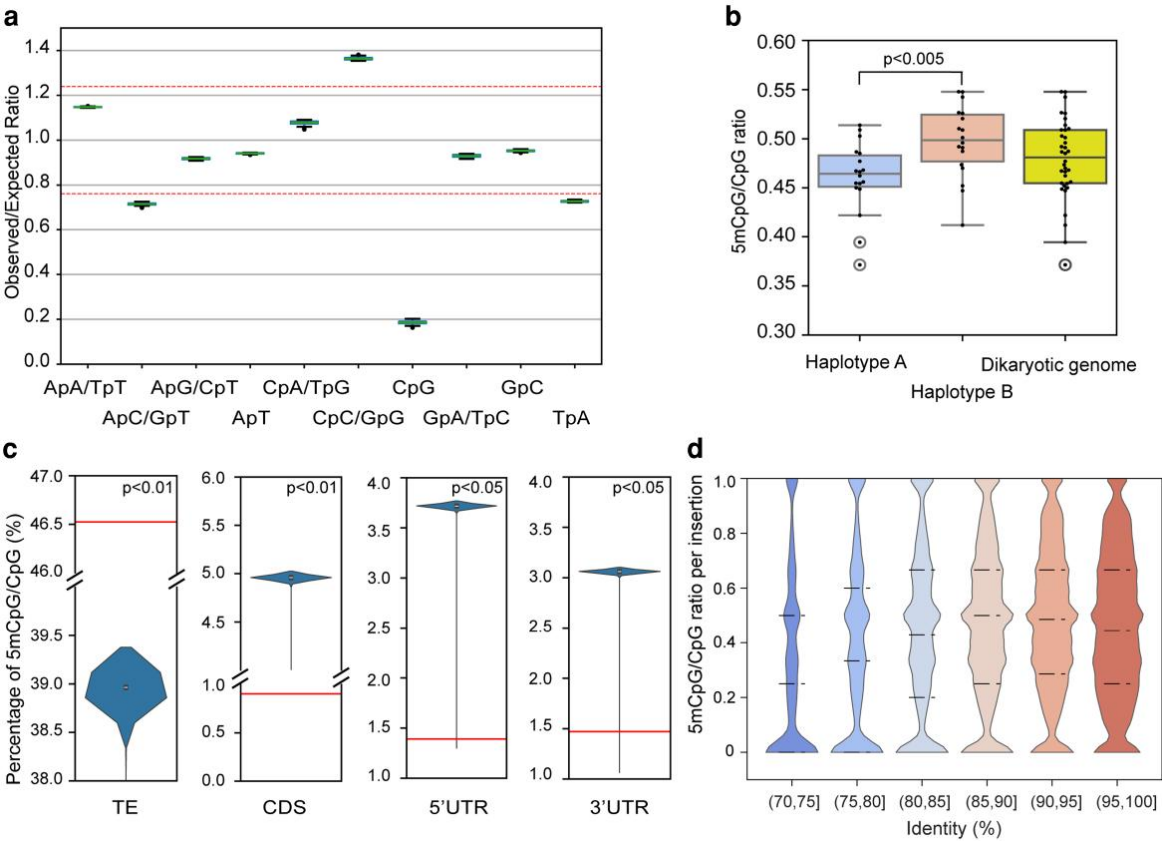
5mCpG methylation is unequally distributed across the *A. psidii* genome and inversely correlated with TE age. a) Dinucleotide observation/expectation (O/E) ratios across chromosomes. Red broken lines at 0.75 and 1.25 represent the threshold of underrepresentation and overrepresentation respectively. b) Ratio of 5mCpG/CpG across chromosomes of haplotype A (blue), haplotype B (orange), and the diploid genome (yellow). The difference between haplotype A and haplotype B is statistically significant (*P* < 0.005). c) Percentage of 5mCpG/CpG overlapping TEs, coding DNA sequence (CDS), 5′ untranslated region (UTR), and 3′ UTR. Violin plots show the distribution of values from permutation tests, while red horizontal lines indicate the observed genome-wide percentage of 5mCpG/CpG in each feature category. d) Ratio of 5mCpG/CpG per insertion of TEs. TEs are categorized into bins based on their sequence identity, with each bin representing a 5% interval ranging from 70% to 100% identity. Dashed lines indicate the 25th, 50th, and 75th percentiles, respectively.

We also examined the genome-wide dinucleotide composition. The genome-wide dinucleotide observed/expected (O/E) ratios show that CpG, ApC (GpT), and TpA are underrepresented, while CpC (GpG) is overrepresented (Fig. 4a). We determined the 5mCpG methylation levels on the remaining CpG sites and revealed that the ratio of methylated CpG sites varies across chromosomes from 0.37 to 0.54 (Fig. 4b). Chromosomes contained in hapB displayed a significantly higher ratio of methylated CpG sites compared to hapA (*P* < 0.005) (Fig. 4b). To investigate whether TEs were preferentially methylated, a permutation test of random occurrence of 5mCpG fractions was performed. The nonrandom occurrence of 5mCpGs suggests that the TEs were hypermethylated (*P* < 0.01) whereas CDS (*P* < 0.01) and UTRs (both *P* < 0.01) are hypomethylated (Fig. 4c). When focusing on TEs, the results revealed that younger TE families displayed significantly higher 5mCpG ratios than older TE families. This suggests that DNA methylation levels play a more important role in TE silencing of younger TE families.

**Fig. 5.**
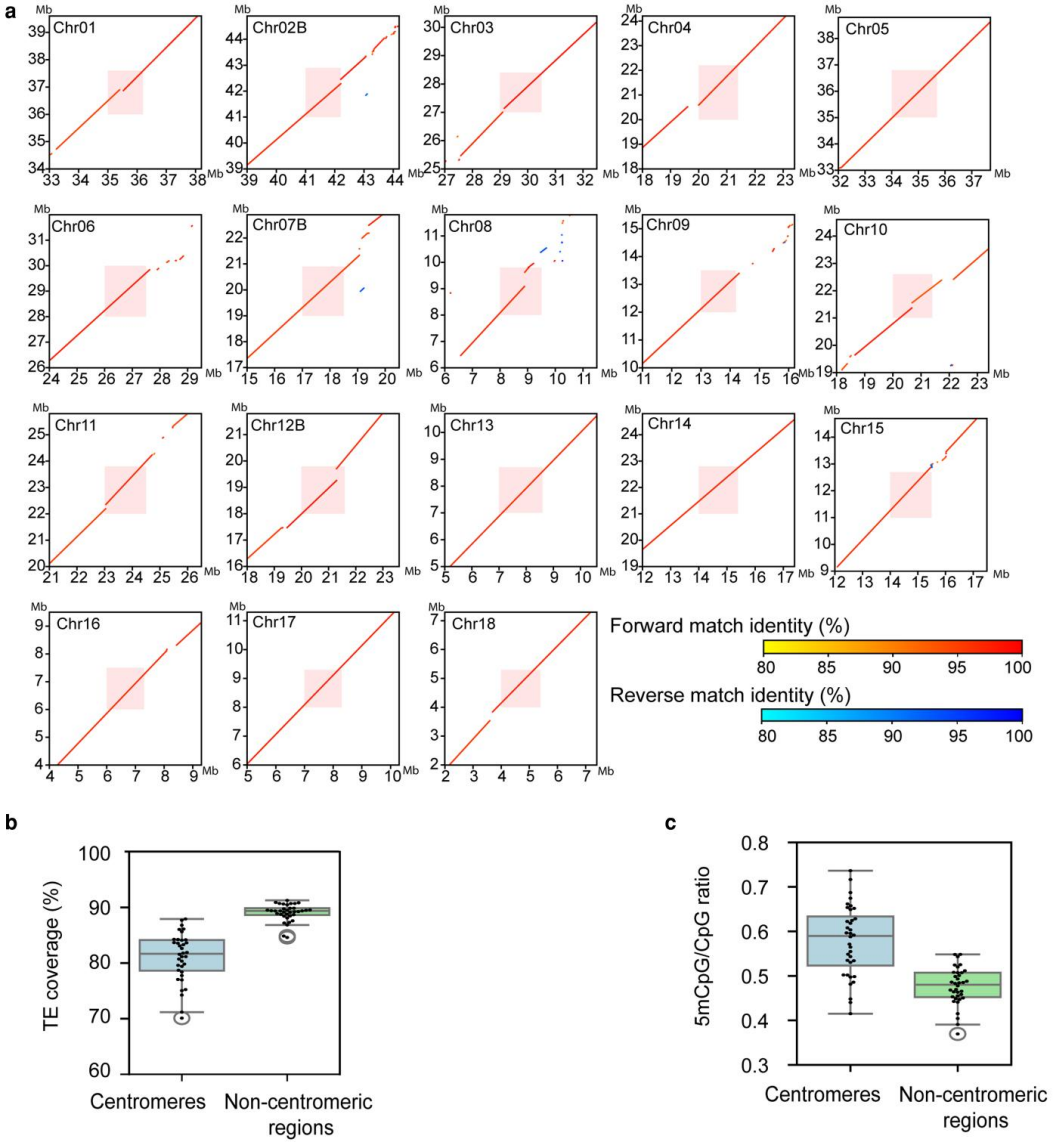
Centromeres are syntenic between orthologs chromosome pairs and hypermethylated. a) Dot plot shows similarity of centromeres and their adjacent regions between haplotypes A and B. The *x* axis represents chromosomes phased in haplotype B, while the *y* axis represents chromosomes phased in haplotype A. Red dots represent regions aligned in the same orientation, while blue dots represent regions aligned in the reverse direction. The positions of the centromeres are highlighted in red. b and c) Comparison of TE coverage b) and the 5mCpG/CpG ratio c) within centromeres and non-centromeric regions. Centromeres showed significantly higher 5mCpG/CpG ratios (*P* < 0.01) b), whereas lower TE density compared to non-centromeric regions (*P* < 0.01) c). Each dot represents the value on a single chromosome.

Next, the centromeric regions of the repeat-rich genome of *A. psidii* were investigated, guided on the classic bowtie structure clearly discernible in the Hi-C contact map obtained from the urediniospore stage (Fig. 2). Analysis of the nucleotide sequence conservation in centromeres between sister chromosomes showed that nearly all sister chromosomes maintained conserved synteny, except chromosome 8 (Fig. 5a). While the centromeric regions were conserved, their flanking areas contained syntenic gaps (Fig. 5a). Given that fungal centromeres are characterized by high TE coverage and cytosine hypermethylation—both important for stability—we analyzed TE density and 5mCpG/CpG ratios. Centromeres showed significantly higher 5mCpG/CpG ratios (*P* < 0.01), but lower TE density compared to non-centromeric regions (*P* < 0.01) (Fig. 5, b and c; Supplementary Fig. 8).

### Detailed MAT locus analysis uncovers a significantly extended PR locus

**Fig. 6.**
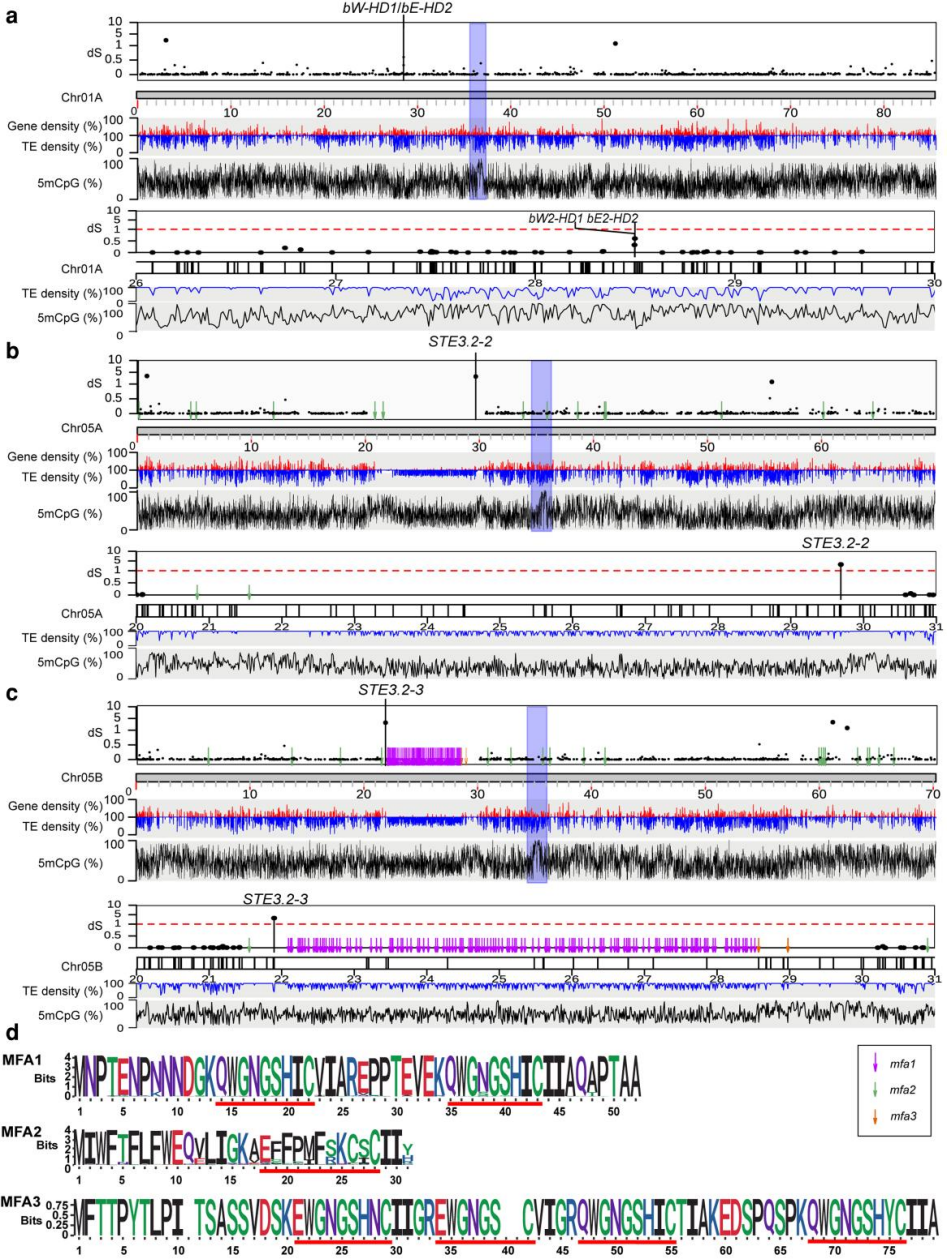
The PR locus is highly enriched in TEs and displays a proliferation of potential MAT pheromone peptide precursor genes. Synonymous divergence values (*d_S_*) for all allele pairs are plotted along chromosomes a) 1A, b) 5A, and c) 5B. In each panel, the top track shows the *d_S_* values of allele pairs along chromosomes, where each dot on the top track represents *d_S_* of a single allele pair. The second, third, and fourth track show the average TEs (“TE”), gene (“gene”) density, and ratio of 5mCpG/CpG (“5mCpG”) along chromosomes in 10 kbp-sized windows, respectively. Predicted centromeric regions are marked with blue shading; predicted mfas are labeled with pink arrows (*mfa1*), blue arrows (*mfa2*), and orange arrows (*mfa3*). The lower tracks provide a detailed zoomed-in view of regions around a) the HD locus and b and c) PR locus, black markers on chromosome tracks represent genes. d) Sequence logos of predicted MFA1, MFA2, and MFA3, with predicted mature peptide pheromone sequences indicated by red underlines. Amino acids are colored based on chemical polarity.

The genome biology of the 2 MAT loci (HD and PR locus) in the new near-T2T genome was also characterized. The HD locus encoding the 2 HD transcription factors (*bW-HD1* and *bE-HD2*) is located on chr1. The PR locus encoding *Pra* pheromone receptor alleles (*STE3.2-2* and *STE3.2-3*) is located on chr5. The overall synteny of the HD locus was conserved between chr1A and chr1B, with no obvious changes in heterozygosity, gene order, and TE density when compared to adjacent regions on the same chromosome (Fig. 6a; Supplementary Fig. 9). This is in stark contrast to the PR locus, which is heavily enriched in TEs, depleted in genes, and extends over ∼10 Mbp (Fig. 6, b and c). The 5mCpG DNA methylation levels do not appear to be elevated at the PR locus. *STE3.2-2* is positioned proximally on the long arm of the chromosome, while *STE3.2-3* appears to be positioned closer to the short arm.

Next, we identified all potential pheromone peptides recognized by the 2 *Pra* receptors. To identify *mfas* in *A. psidii*, we screened ORFs between 90 and 300 base pairs encoding peptides contained “CAAX” motifs. The MFA candidates were classified into 3 categories based on specific sequence features and numbers of tandem repeats: *mfa1*, *mfa2*, and *mfa3*. Manual inspection of tandem repeats revealed the sequence pattern “[Q/E]WGNGSH[X]C” with high frequency among most potential *mfa*1/3s; therefore, this pattern was used for further filtering *mfa*1/3s (Fig. 6d).

In total, 14 copies of *mfa2* on chr5A, 19 copies of *mfa2*, 137 copies of *mfa1*, and 4 copies of *mfa3* on chr5B were identified (Fig. 6d). All *mfa1* and *mfa3* clustered between 22 and 29 Mb of chr5B, which is likely to be the recombination suppressed region of the PR locus containing *STE3.2-3* (Fig. 6c). In contrast, *mfa2* genes were dispersed across both chr5A and chr5B, showing no obvious clustering pattern. Next, MFA coding sequences were further searched in the whole genome and identified an additional 314 copies of *mfa2*s on non-PR locus containing chromosomes.

### Transcriptional regulation reveals 2 waves of expression during early *vs* late plant infection and includes allele-specific expression of CEs

Illumina RNA sequencing was used to understand gene expression of *A. psidii* during infection of *S. jambos* over time. The experimental design included ungerminated urediniospores and 2 to 6 dpi on *S. jambos* (Supplementary Table 5).

**Fig. 7.**
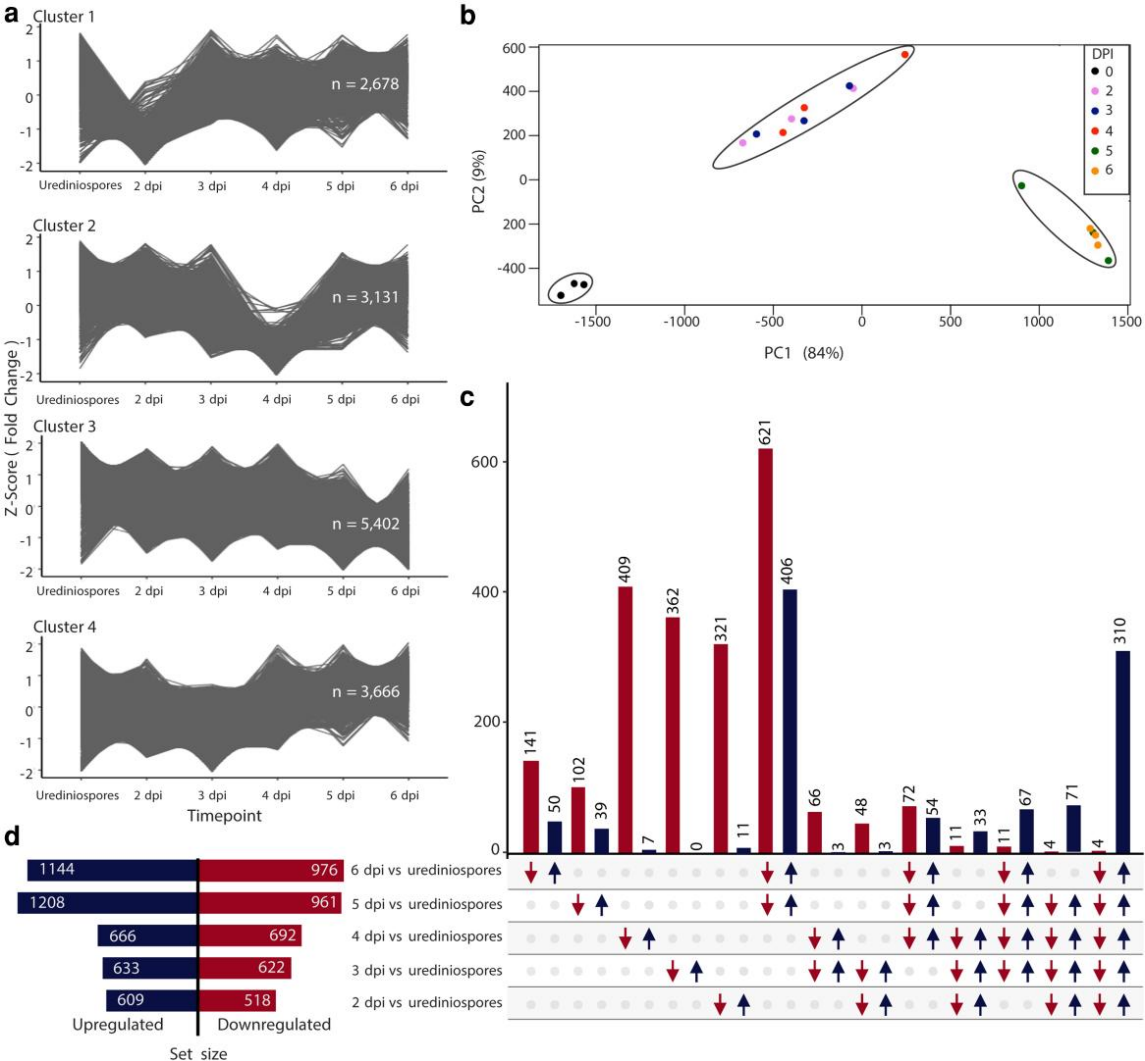
*A. psidii* displays a biphasic transcriptomic reprograming upon infection of *S. jambos* over time. a) *k-*means cluster plots from RNA-seq data analyzed using the TCseq package, utilizing the fold change z-score option for normalization. Each cluster represents distinct expression patterns across the time points. The number of genes that belong to each cluster is highlighted inside the graph. b) Multidimensional scaling plot illustrating the relationships among transcript expressing profiles from 6 different sample groups. c) UpsetR plot showing DEGs specific to each pairwise comparison. The number of upregulated (dark blue bars, arrow directed upward) and downregulated (red bars, arrow directed downward) transcripts is presented on the upper side of each intersection bar. d) Total changes in the *A. psidii* expression profile in planta when compared to resting urediniospores. The total number of DEGs for haplotype A and B, in each pairwise comparison is represented by dark blue (upregulated) and red (downregulated) bars.

We confirmed the accuracy of the RNA-seq analysis and relatedness of biological replicates, using PCA cluster analysis. The analysis suggests that the differential gene expression patterns were strongly linked to sampling time, showing that replicates of earlier time points (2, 3, and 4 dpi) clustered separately from later time points (5 and 6 dpi) and ungerminated urediniospores (0 dpi) (Fig. 7b). This suggests low biological variability among replicates and high degree of similarity in their expression profiles. The first principal component accounts for 84% of the variation between time points, and the second component accounts for 9%.

To explore potential differences in gene regulation during infection, allele-specific expression (ASE) analysis was performed between each time point of infection (2, 3, 4, 5, and 6) in *S. jambos* and the ungerminated urediniospores (0 dpi). In total, 3,767 differentially expressed genes (DEGs) were identified (Supplementary Table 6), of which 1,366 were upregulated in at least one time point in planta relative to ungerminated urediniospores (Fig. 7, c and d). More specific overlapping DEGs were identified in latter time points (5 and 6 dpi) when compared to earlier time points (2, 3 and 4 dpi) (Fig. 7, c and d). Since the focus was the molecular profile of *A. psidii* during interaction with its host, the remainder of this study focused on DEGs that were upregulated in planta. A total of 310 genes were commonly upregulated across all time points, suggesting a common repertoire throughout infection, while 442 genes were upregulated only at 5 and 6 dpi, and 34 genes were commonly upregulated at 2, 3, and 4 dpi (Fig. 7c; Supplementary Table 6). The top upregulated genes in all time points were identified from the DEGs table and resulted in 22 genes, 16 of those classified as CEs (Table 2).

**Table 2.** Candidate effectors are enriched in highly expressed genes in planta.

Gene ID	Chromosome	Annotation	LFC—days post-inoculation vs ungerminated urediniospores				
			2	3	4	5	6
MK676_012155	AU3_HapA_CHR07	Hypothetical protein	15	14	14	17	16
MK676_015034	AU3_HapA_CHR09	Shared candidate effector^a^	16	15	16	14	13
MK676_001717	AU3_HapA_CHR01	Shared candidate effector	14	14	15	14	13
MK676_012151	AU3_HapA_CHR07	Shared candidate effector	13	12	13	15	14
MK676_004721	AU3_HapA_CHR03	Hap.-specific candidate effector	10	11	11	15	15
MK675_016128	AU3_HapB_CHR13	Ubiquitin-like protein atg8	14	14	11	13	12
MK676_017021	AU3_HapB_CHR10	Histone H3.1	14	12	14	12	13
MK676_010149	AU3_HapA_CHR06	Hypothetical protein	14	13	12	9	7
MK676_002979	AU3_HapA_CHR02	Hypothetical protein	14	12	13	10	11
MK675_011626	AU3_HapB_CHR09	Shared candidate effector	13	11	13	14	14
MK676_001658	AU3_HapA_CHR01	Shared candidate effector	11	11	11	14	14
MK675_007778	AU3_HapB_CHR06	Shared candidate effector	10	11	11	14	14
MK676_010164	AU3_HapA_CHR06	Shared candidate effector	10	9	11	14	14
MK675_004123	AU3_HapB_CHR03	Hap.-specific candidate effector	10	10	11	14	14
MK676_009978	AU3_HapA_CHR06	Shared candidate effector	10	9	10	13	14
MK676_023771	AU3_HapA_CHR15	Shared candidate effector	10	10	10	14	14
MK675_014046	AU3_HapB_CHR11	Hypothetical protein	9	9	7	14	14
MK675_000744	AU3_HapB_CHR01	Shared candidate effector	8	11	11	14	14
MK675_017040	AU3_HapB_CHR15	Shared candidate effector	7	10	10	14	14
MK676_025063	AU3_HapA_CHR17	Shared candidate effector	12	10	14	8	10
MK676_019997	AU3_HapA_CHR13	Shared candidate effector	13	11	13	13	12
MK676_005025	AU3_HapA_CHR03	Shared candidate effector	12	12	13	11	10

Top upregulated genes in planta compared to ungerminated urediniospores.

LFC, log-fold change; Hap, haplotype.

^a^Shared candidate effectors are alleles present in both haplotypes.

To obtain biological meaningful information from differential expression results, putative functions were assigned to genes significantly upregulated in planta. Forty-eight percent (657/1366) of all upregulated genes were annotated with GO terms, and/or PFAM terms (Supplementary Table 6 and Fig. 10).

**Fig. 8.**
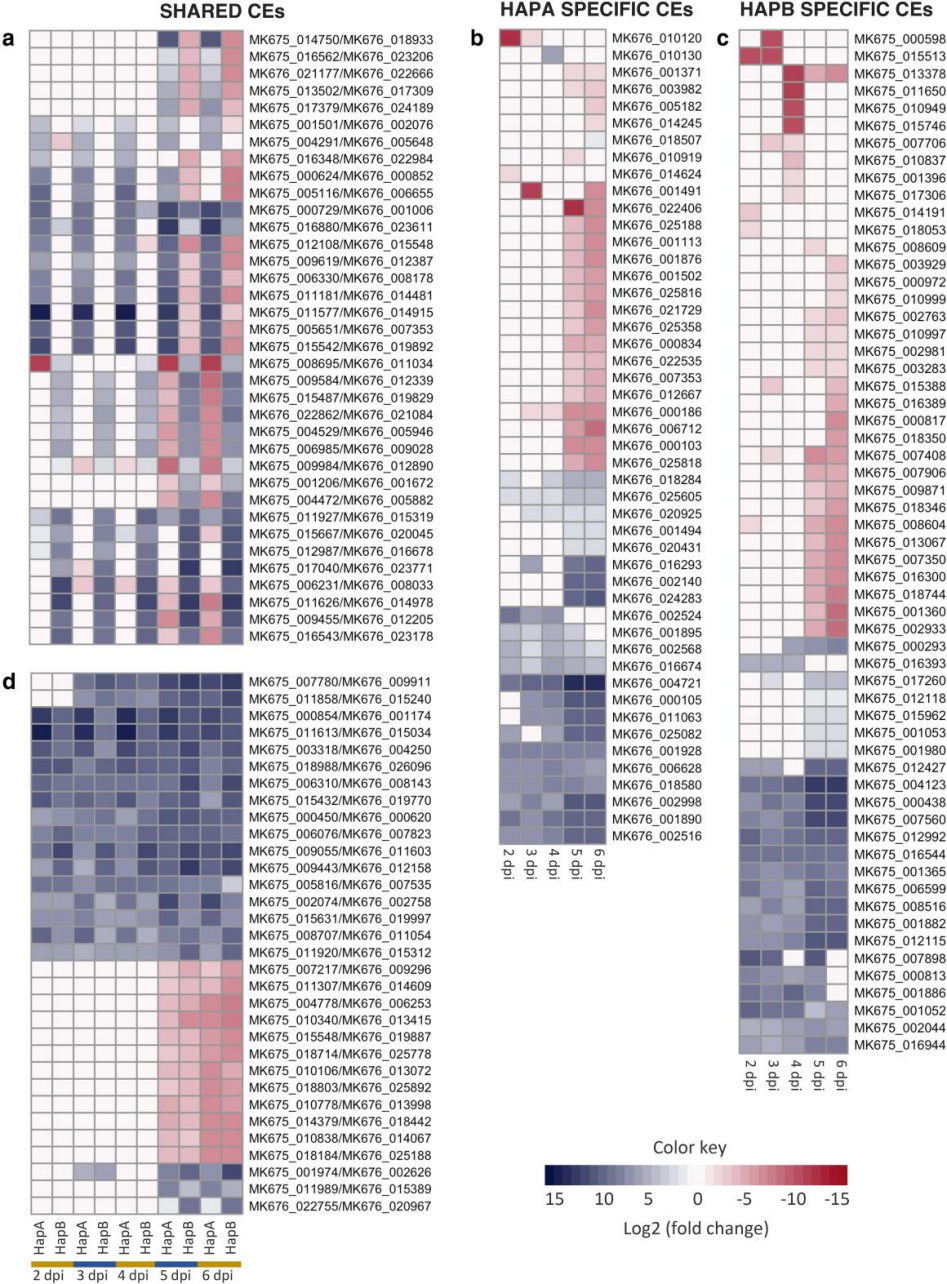
Haplotype-specific expression of candidate effectors (CEs) appears to be common in *A. psidii*. Heatmaps of differentially expressed CEs in planta when compared to ungerminated urediniospores over time. Contrasting expression of alleles in haplotype A (hapA) and haplotype B (hapB) a) and alleles with the same pattern of expression in hapA and hapB b). HapA-specific CEs c) and hapB-specific CEs d). dpi, days post-inoculation.

Plant pathogens typically use secreted effector proteins to facilitate host colonization (Baid et al. 2023). Considering the presence of 2 copies of Chr14 in hapA, for Au3, 1,206 and 979 secreted proteins were identified for hapA and hapB. From those, 283 (23.4%) and 240 (24.51%) are predicted to be CEs based on the evidence of changes in expression during *S. jambos* infection. Most of the CEs were predicted to be secreted to extracellular spaces. Thirty and 25 CEs were hemizygous and only present in hapA or hapB including 2 in the top overexpressed genes in planta (Tables 2 and 3). Notably, 87% of the CEs have alleles present in both haplotypes. Among these shared alleles, only 32 display consistent expression patterns, while the remaining alleles exhibit ASE (Fig. 8; Supplementary Table 7).

**Table 3.** Predicted localization of candidate effectors.

Haplotype	# of Secreted/CEs	Shared				Hemizygous			
		# of CEs	# of Ext.	# of PM	# of Cyt.	# of CEs	# of Ext.	# of PM	# of Cyt.
hapA	1206/283	253	190	13	50	30	23	2	5
hapB	979/240	215	167	6	42	25	14	1	10

Allelic and hemizygous CEs of *A. psidii* v3 genome, haplotype A (hapA) and haplotype B (hapB).

Ext, extracellular; PM, plasma membrane; Cyt, cytosolic.

## Discussion

The pandemic biotype of *A. psidii* exhibits an unusual lifestyle compared to other rust fungi, characterized by an expanding list of host species within the Myrtaceae (Soewarto et al. 2019). This pathogen's dikaryotic nature, combined with its 1 Gb haploid genome size and high repeat content, presents significant challenges for genome assembly (Tobias et al. 2021). These challenges can now be addressed through the integration of advanced sequencing technologies such as PacBio HiFi, ONT ultra-long reads, and Hi-C data (Li and Durbin 2024) permitting deeper investigation into the genome biology and adaptive evolutionary potential of this damaging pathogen. Here, biological approaches and the assembly of a fully phased genome for the pandemic lineage of *A. psidii* were integrated to answer important biological questions.

### Karyotype conservation and genome plasticity in *A. psidii*

Our research shows that *A. psidii* has 36 dikaryotic (n+n) chromosomes consistent with karyotypes of other distantly related rust fungal species (Boehm et al. 1992; Boehm and Bushnell 1992) and despite its phylogenetic placement within the family Sphaerophragmiaceae (Boehm et al. 1992; Beenken 2017). Of the assembled chromosomes, 29 have telomeres at both ends indicating complete chromosomes. Early karyotyping efforts underestimated chromosome numbers in rust fungi, reporting *n* = 3 to 6 (Valkoun and Bartoš 1974; Goddard 1976) due to small size and challenges in arresting the metaphase state in biotrophs. Subsequent studies on rust species, including *M. lini* (Boehm and Bushnell 1992) and *P. graminis* f. sp. *tritici* (Boehm and Bushnell 1992), consistently identified a haploid chromosome number of *n* = 18. These findings have been recently further confirmed with new technology that has enabled phasing and scaffolding of genomes for *P. triticina* (Duan et al. 2022), *P. striiformis* f. sp. *tritici* (Schwessinger et al. 2022; Tam et al. 2025), *P. polysora* f. sp*. zeae* (Liang et al. 2023), and *M. lini* (Sperschneider et al. 2025). This suggests that the chromosome numbers are highly conserved among the rust fungi which diverged about 85 mya (Aime et al. 2018). Our further comparisons of the conservation of chromosomal locations of shared BUSCOs revealed that these are clearly conserved between *A. psidii* and the 2 other investigated rust fungi *M. lini* and *P. graminis* f. sp. *tritici*. Outside of the order Pucciniales, this 1:1 chromosomal pairing appears to be eroded as the comparison of *A. psidii* with a *U. bromivora* and *S. panici-leucophaei* clearly show. In the future, more quantitative measures of synteny, like syntenic correlation, which can quantify the extent to which orthologous genes deviate from independent random distribution across the genomes of 2 species (Housworth and Postlethwait 2002), will provide a more detailed insight in synteny conservation over evolutionary distance within Pucciniales and beyond.

In *A. psidii*, the large genome and chromosome size are primarily attributed to the amplification of repetitive DNA sequences, including TEs (Tobias et al. 2021). The large genome of *A. psidii* prompted our cytological investigations based on the expectation that chromosomes are likely to be more visible at metaphase state than in cereal rust fungi. Our cytological study found that *A. psidii* chromosomes can be clearly separated into 2 distinct size classes of 12 large and 6 small chromosomes, resulting in a bimodal karyotype, which is an interesting feature often seen in birds (Waters et al. 2021). This marks the first cytological study of *A. psidii* chromosomes, with the sizes aligning well to the distribution determined from sequencing analysis, suggesting that the karyotype reflects the fungal genomic structure. Further comparative genomic work among rust fungi covering the whole phylogeny will help to answer questions on karyotype conservation and genome evolution in response to continuous host adaptation.

### Unequal chromosome numbers within the *A. psidii* haploid nuclei

Dikaryotic rust fungal evolution is characterized by extensive mitotic division leading to high levels of heterozygosity (Duan et al. 2022). The observation of uneven chromosome counts in 2 haplotypes have been previously reported in *P. graminis* f. sp. *tritici* 21-0, where both copies of chr11 were assigned to the same haplotype based on Hi-C analysis (Li et al. 2019). Since nuclei fusion is absent during asexual reproduction of rust fungi (Savile 1939), this “chromosome-swap” is likely to occur during meiosis. Mis-segregation events during this process can result in both copies of chr14 being directed to the same nucleus. Since mitosis lacks nuclei fusion, it is plausible that the chromosome swap can be inherited asexually. If such an event initially generated haploid spores lacking chr14, survival until dikaryon formation would imply that this chromosome does not encode essential functions. Instead, as suggested for accessory or conditionally dispensable chromosomes in other fungi, its gene content may be more related to adaptive traits, such as host interaction or virulence, rather than core cellular processes (Croll and McDonald 2012). Our annotation data from chr14 revealed the presence of several genes typically categorized as housekeeping, such as histone H3, cell cycle regulators, ubiquitin proteases, and DNA repair helicases. While this finding appears contradictory to the meiotic mis-segregation hypothesis, a comparative analysis of both haplotypes demonstrated that these functional genes are not unique to chr14 and that paralogous copies are present on other chromosomes. The presence of core functional gene redundancy could support the hypothesis that chr14 is dispensable or is involved in virulence. It is unclear if this genome plasticity may fulfill a similar function to aneuploidy in yeast species, such as *Saccharomyces cerevisiae* and *Candida albicans*, where different chromosomes contribute to adaptive phenotypes (Rustchenko 2007; Rancati et al. 2008; Sah et al. 2021) or whether our data accords with ascomycete fungal species also described with unequal haploid chromosome numbers (Xu et al. 2025). The mechanisms underlying this unequal distribution of chromosomes while still maintaining spore viability are not yet known (Xu et al. 2025).

Our work also shows greater homology between predicted proteins for chr14 compared to all the other chromosomes (Supplementary Fig. 5). This greater homology may be explained by homologous recombination (Moynahan and Jasin 2010; Ip et al. 2020) during extended non-sexual reproduction, whereby mutations are repaired based on the paired chromosome, while the other chromosomes, in separate nuclei, gain mutations and diverge. A speculative explanation is that a meiotic event in a precursor biotype to the pandemic developed this nuclei chromosome arrangement and asexual proliferation led to the dominant pandemic spore type that has expanded globally infecting a broader host range. The finding of a Brazilian biotype closely related to the pandemic *A. psidii* (Luo et al. 2025) may present a useful opportunity to further investigate this phenomenon. An alternative explanation could involve a mis-segregation event during mitotic division followed by selection or random drift on isolates that carry unequal chromosome numbers in their nuclei.

Overall, it is noteworthy that chr14 of *A. psidii* and chr11 of *P. graminis* f. sp. *tritici* are not orthologous, as would be expected if the phenomenon originated during mitosis. Additionally, unequal sorting of haploid chromosomes has been validated in ascomycetes (Xu et al. 2025), suggesting that similar mechanisms may be at play in rust fungi, potentially influencing their adaptability and evolutionary dynamics.

### DNA methylation plays a role in TE silencing in *A. psidii*

It is widely accepted that the CpG depletion of many animal genomes is caused by methylation and mutational loss of cytosines at CpG site (Bird 1980; Bird and Taggart 1980; Simmonds et al. 2013; Ip et al. 2020). In many genomes, including the 1 Gb *A. psidii* genome compared to other rusts, there is a negative correlation between genome size and the O/E ratio of the CpG dinucleotides, suggesting that the suppression of TE activity by the 5mCpG deamination is essential for their long-term accommodation of TE's in the host genome (Zhou et al. 2020).

We observed significant genome-wide CpG depletion, likely to be the result of deaminated 5mCpG within TEs. Previous analysis on the v1 genome indicated that low GC content is unlikely to result from RIP mutation as *A. psidii* displayed insignificant RIP indices and an absence of genes associated with RIP mutation mechanisms. Hence the low GC content was hypothesized to be the consequence of genome-wide deamination of methylated cytosines which cause C-to-T transition mutations (Tobias et al. 2021). The current findings provide further support for this hypothesis, as 5mCpG/CpG ratio and GC content is higher in younger TE insertions than in aged insertions. This suggests that DNA methylation is the initial mechanism that silences younger TEs, and deamination and C-to-T are secondary TE silencing mechanisms (Ying et al. 2022). Additionally, the preferential cytosine methylation of TEs compared to genic regions of the genome highlights the role of DNA methylation in silencing these elements, contributing to overall genome stability.

Consistently, centromeres in rust fungi have been reported to be hypermethylated (Sperschneider et al. 2021; Tam et al. 2025), which is hypothesized to maintain chromatin stability during nuclear division and chromosome segregation (Hernández-Saavedra et al. 2017). The identification of hypermethylated centromeres in *A. psidii* is consistent with these previous observations. Unlike other rust fungi, we did not observe TE coverage enrichment in centromeric regions compared to non-centromeric regions. It is currently unclear if this is a biological feature or a technical artifact. It could be that we missed centromeric TEs by employing the previously generated de novo TE database based on genome v1, which might have lacked centromeric sequences (Tobias et al. 2021). Yet careful follow-up investigation of centromeric regions did not reveal obvious repetitive sequences in centromeric regions of genome v3. Hence the under-annotation of TEs in centromeres might also suggest that *A. psidii* centromeres are not enriched in TEs. In future studies, performing centromere analysis with a TE database built on the updated genome and including additional *A. psidii* genomes might resolve this outstanding question.

### Signals of extensive genomic degeneration around the *A. psidii* PR locus and significant amplification of MFA peptide precursor ORFs

We performed detailed analysis of the *A. psidii* MAT loci. Our analysis further supports the findings of Ferrarezi et al. (2022) that *A. psidii* has a tetrapolar mating system. Investigation of the full HD locus reveals minimal genomic degeneration while the PR locus exhibit significant degeneration, creating large syntenic gaps. These results are consistent with previous findings for cereal rust fungi (Henningsen et al. 2024; Luo et al. 2024).

The identification of extensive copies of *mfas* is unexpected, since only 2 to 3 copies of *mfas* have been identified in other rust fungi (Cuomo et al. 2017; Henningsen et al. 2024; Luo et al. 2024). These *mfa* duplications and translocations are likely related to TE insertions. Moreover, the high conservation of tandem repeat sequences within *mfa*1/3 across rust fungi, despite substantial evolutionary divergence, strongly suggests potential purifying selection on these motifs due to their important function for *STE3.2-2* and *STE3.2-3* pheromone receptor recognition. Additionally, we identified that the PR locus is significantly extended to close to 10 Mb in size which is mostly driven by repeat expansions and likely linked to recombination suppression. At the same time, 5mCpG methylation appears not to be enriched when compared to the rest of the genome and the PR locus in *P. striiformis* f. sp. *tritici* (Tam et al. 2025).

Mechanisms involved in meiotic recombination suppression in the sex or MAT regions across organisms include epigenetic modifications, such as DNA cytosine methylation in *Arabidopsis thaliana* (Ge et al. 2024) and histone H3K9 methylation at the MAT locus in fission yeast, which lacks DNA methylation (Oh et al. 2022). Given the sequence divergence between the 2 haplotypes of the PR locus, it is likely that sequence heterogeneity shaped by TE insertions inhibits recombination activity at this locus in the absence of DNA methylation.

### The pandemic biotype of *A. psidii* displays uniform infection dynamics across distantly related plant species

The pandemic lineage of *A. psidii* is linked to the rapid spread and dissemination of the pathogen worldwide (Granados et al. 2017; Stewart et al. 2017; Carnegie and Pegg 2018; du Plessis et al. 2019; Ruffner et al. 2024). This widespread distribution is likely attributable to its polyphagous nature, which allows it to infect a broad range of host species (Soewarto et al. 2019). In contrast, the pathogen demonstrates a higher level of host specialization in its center of origin (Graça et al. 2013; Stewart et al. 2017; Morales et al. 2024; Luo et al. 2025). This duality, where the pathogen thrives in its invasive range globally while maintaining specialization in its native range, highlights the complex ecological dynamics at play and underscores the need for further research into its adaptive strategies.

Studies examining the interaction of *A. psidii* with individual host species have reported vastly different infection time scales on the same host in different laboratories and on different host species across different studies (Glen et al. 2007; Beresford et al. 2020; Degnan et al. 2023). For example, infection dynamics of the susceptible species *S. jambos* ranged from as short as 6 d (Beresford et al. 2020; Degnan et al. 2023) to 12 d (Glen et al. 2007) for the completion of the infection cycle. Our research work shows that the pandemic biotype of *A. psidii* displays very consistent infection dynamics across 4 different host species, despite being separated by 65 mya of evolution (Biffin et al. 2010; Grattapaglia et al. 2012), when using the same infection conditions. This finding emphasizes the importance of more in depth understanding of infection mechanisms on different host species to develop effective management and biosecurity strategies.

### Allele-specific gene expression analysis provides evidence for identifying CEs

While most of the studies investigated the genetic mechanisms of defense of host species against *A. psidii* infection (Hsieh et al. 2018; Tobias et al. 2018; Soewarto et al. 2019; Santos et al. 2020; Swanepoel et al. 2021; Frampton et al. 2024), only 2 studies have examined the molecular genetics of the fungus during the infection of susceptible hosts (Swanepoel et al. 2021; Frampton et al. 2024), both of which used the early version of the *A. psidii* genome (Tobias et al. 2021). Here, we present the first ASE analysis of *A. psidii* over the course of infection in a susceptible host.

The patterns of gene expression clustered into 3 distinct groups: ungerminated urediniospores, early infection stage (2 to 4 dpi), and late infection stage (5 to 6 dpi). These patterns are like those observed in the asexual reproduction of *P. striiformis* f. sp. *tritici* (Schwessinger et al. 2018; Tam et al. 2025). The increase in the number of *A. psidii* DEGs in the late infection stages was also previously reported in the interaction of *A. psidii* with susceptible *E. grandis* and may be attributed to the lack of host defenses, which allows the fungus to reproduce more effectively compared to a resistant host (Schwessinger et al. 2018; Swanepoel et al. 2021).

The early upregulation of CEs from 2 to 4 dpi coincides with the latency phase of the disease (before sporulation), while the overexpression of effectors at 5 dpi coincides with the appearance of symptoms. At 6 dpi, several different patterns were observed: some CEs that were previously downregulated were either upregulated or downregulated to a greater extent, while those CEs that were previously upregulated were either downregulated or maintained their upregulation. This time point coincides with the first appearance of sporulating lesions, marking a significant transition in the infection cycle. Interestingly, 30 and 25 CEs were only present in hapA or hapB respectively, including 2 of the top overexpressed genes in planta. Notably, 13 of the differentially expressed CEs (1 haplotype specific and 12 shared) matched 7 CEs (Tobias et al. 2021) previously reported as expressed in planta (Frampton et al. 2024) (Supplementary Table 7), reinforcing the consistency of part of the repertoire identified across independent studies.

Among the overexpressed genes, the majority possess alleles in both haplotypes, with most exhibiting direction-biased expression favoring one allele over the other. In contrast, only a small subset of genes showed consistent expression pattern in both haplotypes. ASE may arise from various genomic and epigenomic mechanisms, including somatic copy number alterations (deletions or duplications), gene mutations, epigenetic modifications in promoter regions, and cis-acting regulatory mutations that influence transcriptional outcomes (Sen et al. 2022). In plants, 2 categories of ASE genes have been reported: consistent ASE genes, which ASE exhibit bias toward 1 parental allele across conditions, and inconsistent ASE genes, which ASE display bias toward different parental alleles under varying conditions (Shao et al. 2019; Zhan et al. 2024). The latter likely represents adaptive regulation, allowing plants to adjust allelic expression for better response (Shao et al. 2019; Zhan et al. 2024). Since the expression difference caused by ASE may result in phenotypic variation (Shao et al. 2019), future experimental study to verify the CEs might allow better understanding of virulence of *A. psidii*.

## Supplementary Material

jkaf255_Supplementary_Data

## Data Availability

Genome assembly and raw sequencing data have been deposited in NCBI under BioProject accession PRJNA810572 and PRJNA810573, and details of transcriptome data are available in Supplementary Table 1. The functional annotations and TE annotations are publicly available through Zenodo (DOI: 10.5281/zenodo.15522519). Codes used for data analysis are available at https://github.com/ZhenyanLuo/Au3_genome_manuscript.git. Supplemental material available at *G3* online.
