# Supplementary material for: Host adaptation and genome evolution of the broad host range fungal rust pathogen, Austropuccinia psidii
Source: G3 (Bethesda). 2025 Oct 28;16(1):jkaf255. doi: 10.1093/g3journal/jkaf255 (PMC12774611; doi:10.1093/g3journal/jkaf255)
Supplement: jkaf255_Supplementary_Data [file jkaf255_supplementary_data.zip › Supplemental_Material_G3-2025-406293.docx]

**Supplementary methods**

**Scanning Electron Microscopy (SEM)**

Urediniospore suspensions of *A. psidii* Au3 isolate were prepared using 5-day old spores at a concentration of 106 spores/mL-1. Three young *S. jambos* leaves (located at the second internode starting from the apex from the plant) were detached from three different plants, totalizing 9 leaves. Three areas of 0.1 cm2 on the abaxial surface of each leaf were marked and inoculated with 30 µL of urediniospores suspension. The inoculated leaves were incubated at 23 °C, in the dark, inside large Petri dishes with moistened filter paper to maintain high relative humidity for 24 h. This experiment was repeated twice.

Marked samples were collected 24 hours post-inoculation (hpi), fixed and processed as described previously (13). The leaf samples were mounted onto 12.5 mm aluminum stubs with double-sided carbon tape. Mounted samples were then coated with platinum in a Emitech K550 sputter coater. A Zeiss UltraPlus FESEM microscope was used (accelerating voltage of 3kV, working distance 15 mm and magnification 500 X) to obtain micrographs of the samples.

**Confocal Laser Scanning Microscopy (CLSM)**

To characterize *A. psidii* infection and colonization processes inside of S. jambos leaves, infections were performed as described in the methods section 2.1. Leaf samples of approximate size 0.5 cm2 were collected at 3, 6 and 9 dpi, and fixed and processed as described previously for the same pathosystem (13). A Leica Stellaris 8 fluorescence microscope was used to obtain micrographs of the abaxial surface of leaf samples. Samples were placed on microscope slides and covered with a drop of 25 % glycerol. The Alexa Fluor 488 conjugate was excited, and fluorescence was detected between 503-549 nm. Calcofluor white counter staining was excited at 360 nmm and detected around 450 – 473 nm. The ImageJ software was used to obtain maximum intensity Z-projections (128).

**mtDNA data extraction and assembly**

Screening of the initial haploid genome output hapA of Hifiasm identified scaffolds corresponding to partial mitochondrial genomes. HiFi reads were mapped to the full hapA assembly with minimap2 v.2.22 (59) and reads that mapped to mtDNA scaffolds were extracted using SAMtools v.1.15 (60). mtDNA reads were assembled with Hifiasm v.0.16.1 (58) and a 94.5 kb circular contig representing the mtDNA was identified. Error-correction was performed by mapping reads back onto a double-copy draft mtDNA using minimap2 v.2.22 (59) and polishing with HyPo v.1.0.3 (https://github.com/kensung-lab/hypo). A highly uneven depth profile was identified, raising concerns that the assembled reads included both pure mtDNA and nuclear mitochondrial insertions (NUMTs). A subset of mtDNA reads was extracted by searching the double-length draft mtDNA onto all 10+ kb HiFi reads and extracting all reads with 99 %+ read coverage by mtDNA using GABLAM v.2.30.5 (129) wrapping BLAST+ v.2.11.0 (71). This subset was reassembled with Hifiasm and a new 94.5 kb circular contig representing the mtDNA was identified. A double-copy version of this contig was polished with Hypo v.1.0.3 using only the “pure” 10 kb reads and re-circularised to have the same starting position as published *A. psidii* mtDNA sequence NC_044121.1 (130). The assembled mitochondrial genome was annotated with GeSeq (131) web Browser (https://chlorobox.mpimp-golm.mpg.de/geseq.html), using the NC_044121.1 mtDNA as a reference sequence (130).

**Supplementary Figures Captions**

**Figure S1: Infection symptoms on abaxial side of four myrtaceae host plants**

(A-D) shows Infection symptoms developed on abaxial side of infected leaves between 2-/3- and 9-days post inoculation (dpi) for (A) *Syzygium jambos*, (B) *Leptospermum scoparium*, (C) *Melaleuca quinquenervia* and (D) *Melaleuca alternifolia*. Scale bars represent 1 cm respectively.

**Figure S2: Microscopic analysis of *Austropuccinia psidii* infection structures** (A) A germinating *Austropuccinia psidii* urediniospore forming a germ tube and appressorium 24 hours post inoculation. The black scale bar represents 10 µm. (B) Representative scanning electron micrographs of *A. psidii* infection structures at 24 hours post inoculation on *Syzygium jambos* abaxial leaf surfaces showing germination and formation of appressoria. The white scale bar represents 20 µm. (C) Representative fluorescence micrographs showing *A. psidii* development in *S. jambos*, that is at a similar stage in *Melaleuca alternifolia* and *M. quinquenervia* at 3-, 6- and 9-days post inoculation (dpi). At 3 dpi, germinated spores have started growth inside the leaf and formed the first few haustoria in all species. At 6 dpi, uredinia has been formed but has not erupted yet. At 9 dpi, fungal pustules have clearly formed and uredinia has sporulated. The fungal structures were stained using Alexa Fluor-488-WGA dye shown in green, and plant cells shown in red were stained using Calcofluor-white dye. The lower epidermis of the leaf was analyzed under the microscope. The scale bar represents 20 µm. (h, haustorium; s, spore; u, uredinia; sp u, sporulating uredinia).

**Figure S3: Cytology of *Austropuccinia psidii* chromosomes***.* A-D show four different metaphase cells. Chromosomes were counterstained with 4’,6-diamidino-2-phenylindole (DAPI). Chromosomes form a bimodal karyotype, with large chromosomes with clear primary constriction and two chromosome arms, and small chromosomes without visible primary constriction. Bars equal 2 μm.

**Figure S4: Repeat content of the *Austropuccinia psidii* genome v3.** Bar plot shows coverage of repeats on different chromosomes in superfamily levels. Coverages of transposable elements (TEs) in different superfamilies are ordered by total coverage from high to low, colours represent different superfamilies, question marks in legends represent unclassified TEs.

**Figure S5: Orthologous chromosomes of *Austropuccinia psidii* are mostly syntentic** Syntenic relationship between the 18 orthologous chromosomes of *A. psidii* are shown. Pink lines connect genes with more than 70 % identity in their encoded protein sequences, with alignments covering at least 50 % of both the query and reference sequences. Blue lines and black lines above and below chromosome tracks represent ratio of 5mCpG/CpG and transposable element (TE) density in 10kb window size respectively. Centromeric regions of each chromosome are shaded in blue on chromosome tracks.

**Figure S6: Orthologous chromosomes of *Austropuccinia psidii* are mostly syntentic** Dot plots show the sequence conservations between the 18 orthologous chromosomes. The x-axis represents chromosomes phased in haplotype B, while the y-axis represents chromosomes phased in haplotype A. The vertical and horizontal grid lines represent boundaries between chromosomes. Red dots represent regions aligned in the same orientation, while blue dots represent regions aligned in the reverse direction. Chromosome 14AB was placed in haplotype B for better comparison.

**Figure S7: Nucleotide composition of transposable elements found in the *Austropuccinia psidii* genome v3** (A) GC content (%) per insertion of transposable elements (“TEs”). (B) CpG counts per 1 kbp of each insertion of TEs. TEs are categorized into bins based on their sequence identity, with each bin representing a 5 % interval ranging from 70 % to 100 % identity. Dashed lines indicate the 25th, 50th and 75th percentiles, respectively.

**Figure S8: Centromere composition analysis.** Karyoplots show the compositions of the 36 centromeres including 2 Mb flanking regions*.* Orange lines above the chromosomes represent GC content (%) in 10 kb window size. The first track below the chromosome track shows the gene coverage (%) (red) and the transposons coverage (%) (blue) in 10 kb window size. The second track represents the ratio of 5mCpG to CpG. Centromeric regions are highlighted with blue boxes.

**Figure S9: Detailed analysis of the *Austropuccinia psidii*** **HD locus** Synonymous divergence values (d_S_) for all allele pairs are plotted along chromosomes 1B. In each panel, the top track shows the d_S_ values of allele pairs along chromosomes, where each dot on the top track represents d_S_ of a single allele pair. The second, third and fourth track show the average transposable elements (“TE”), gene (“gene”) density and ratio of 5mCpG/CpG (“5mCpG”) along chromosomes in 10 kbp-sized windows respectively

**Figure S10: Functional enrichment analysis of *in planta* differentially expressed genes**. The figure shows a dotplot of Pfam terms which are significantly (p.adjust < 0.05) over-represented in DEGs of at least one comparison. The dot size represents the relative proportion of DEGs whereas colors represent p.adjust values.
