# Supplementary figures and images for: Host adaptation and genome evolution of the broad host range fungal rust pathogen, Austropuccinia psidii
Source: G3 (Bethesda). 2025 Oct 28;16(1):jkaf255. doi: 10.1093/g3journal/jkaf255 (PMC12774611; doi:10.1093/g3journal/jkaf255)

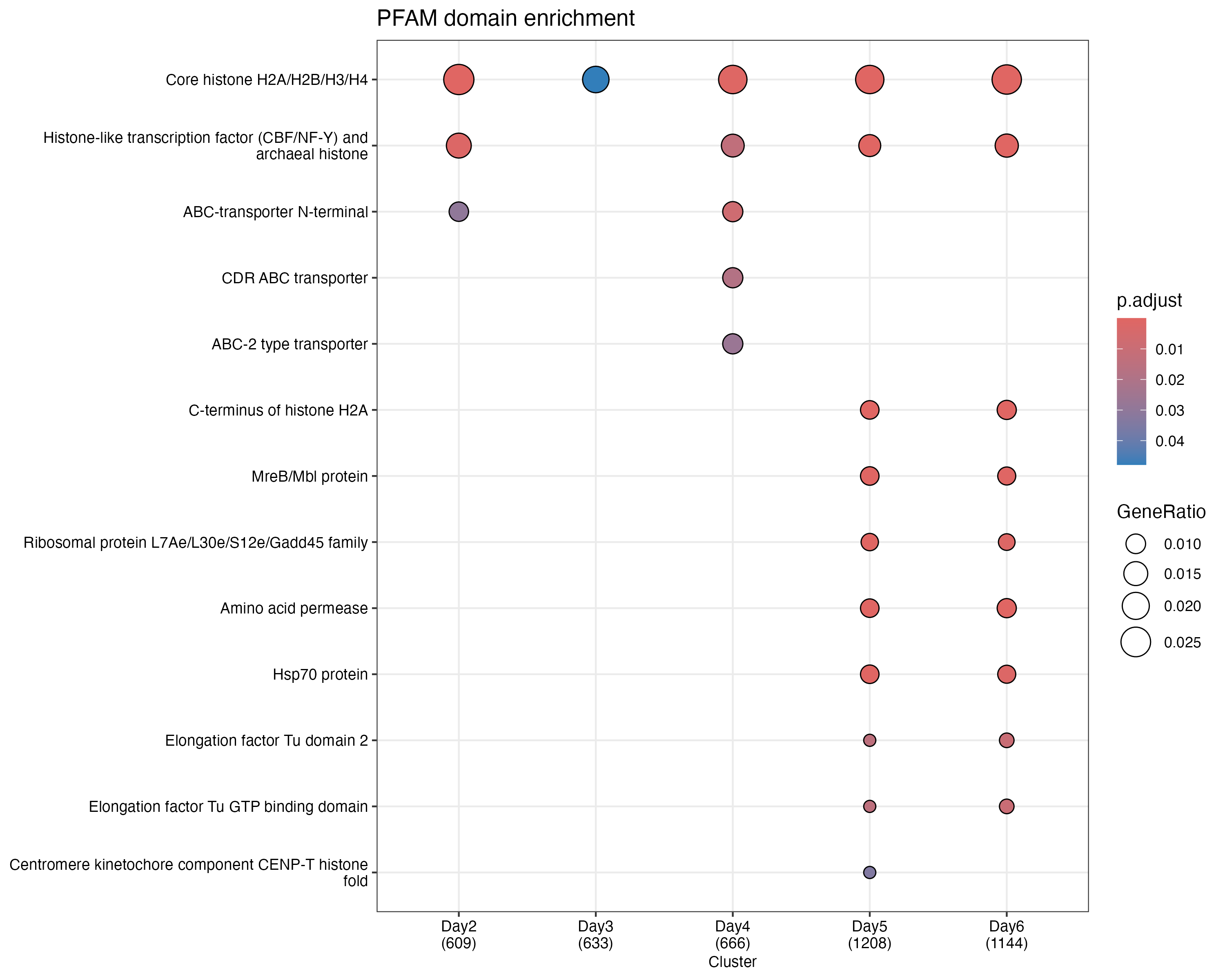

Supplement: jkaf255_Supplementary_Data [file jkaf255_supplementary_data.zip › Supplemental_Figure_10_G3-2025-406293.tif]

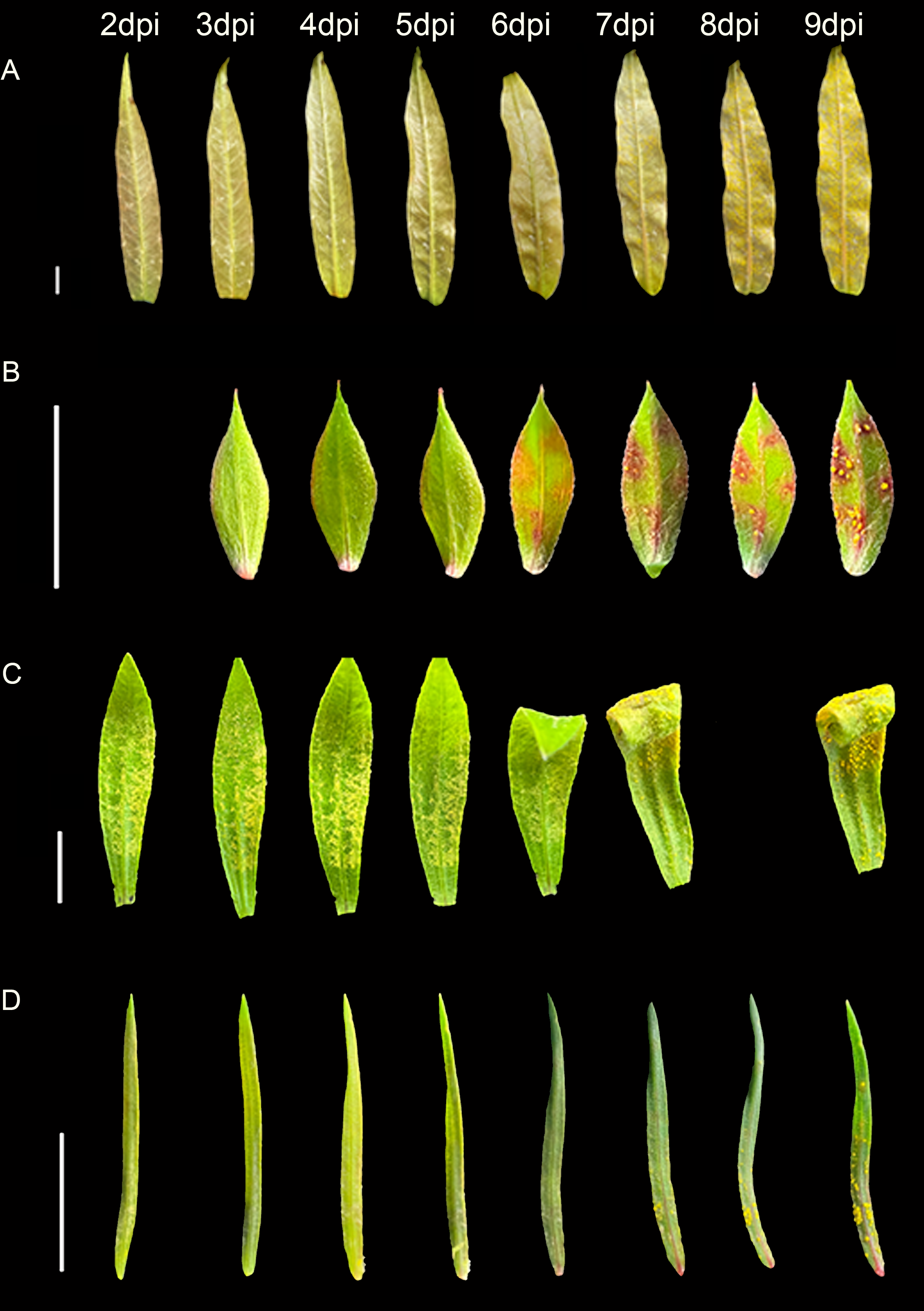

Supplement: jkaf255_Supplementary_Data [file jkaf255_supplementary_data.zip › Supplemental_Figure_1_G3-2025-406293.tif]

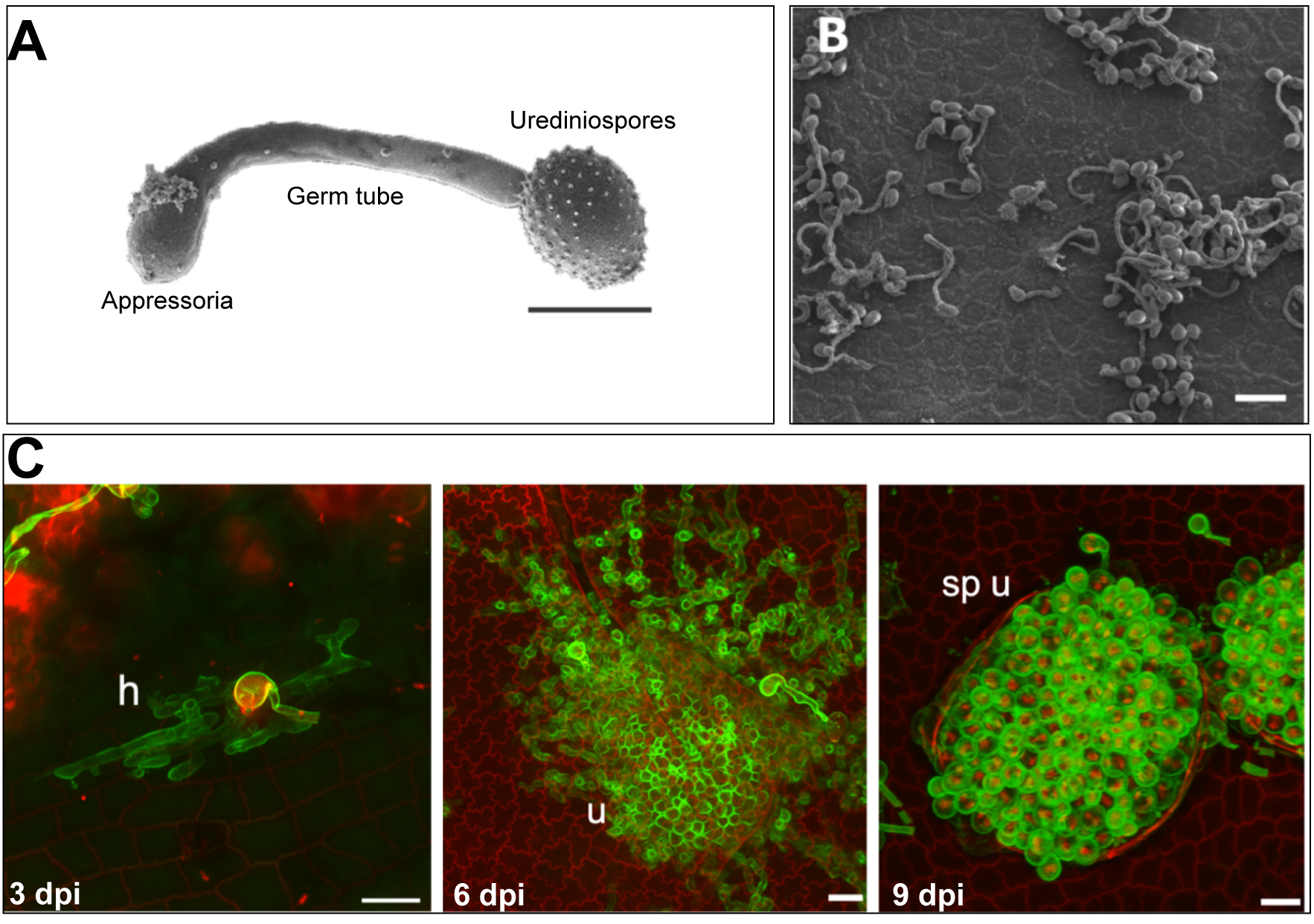

Supplement: jkaf255_Supplementary_Data [file jkaf255_supplementary_data.zip › Supplemental_Figure_2_G3-2025-406293.tif]

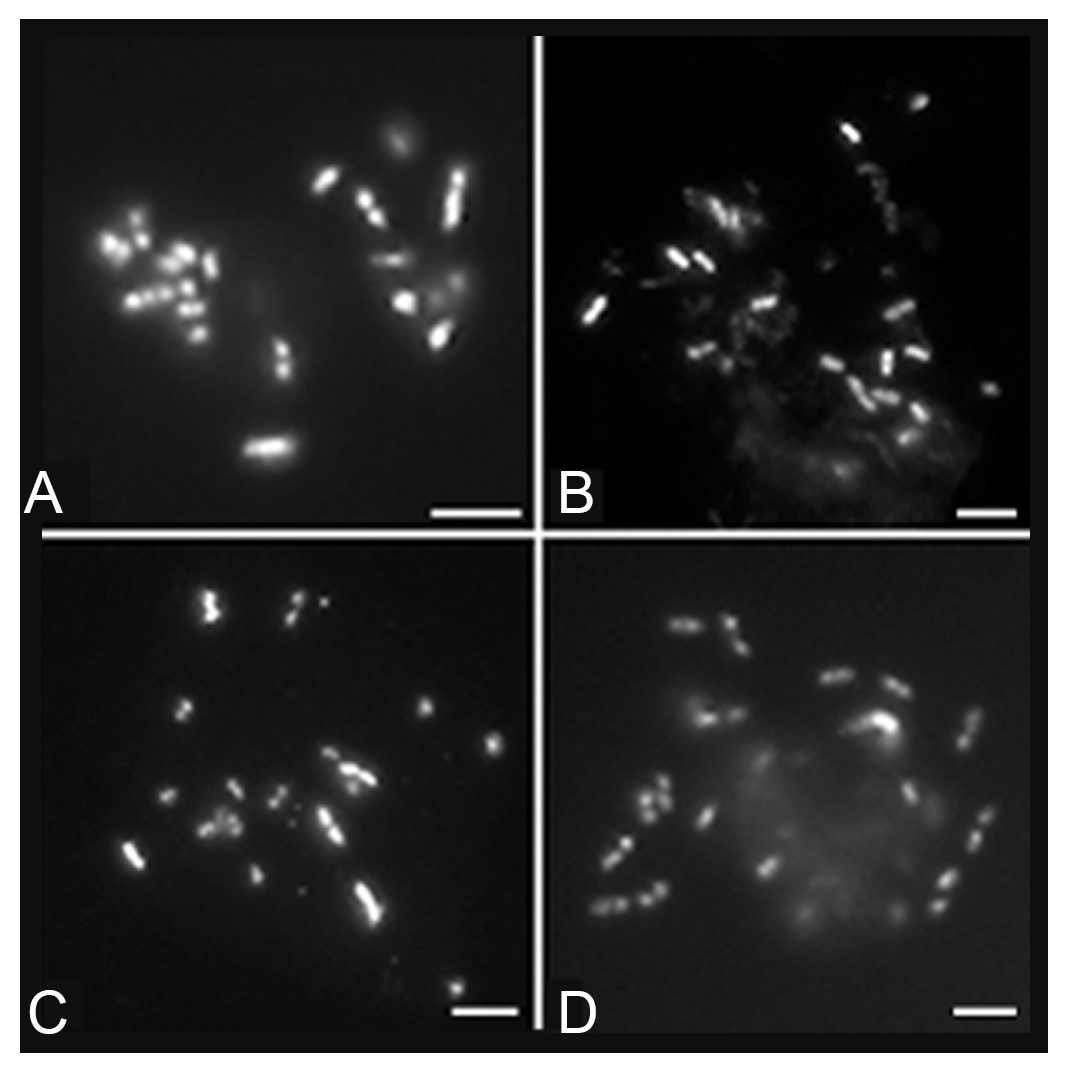

Supplement: jkaf255_Supplementary_Data [file jkaf255_supplementary_data.zip › Supplemental_Figure_3_G3-2025-406293.tif]

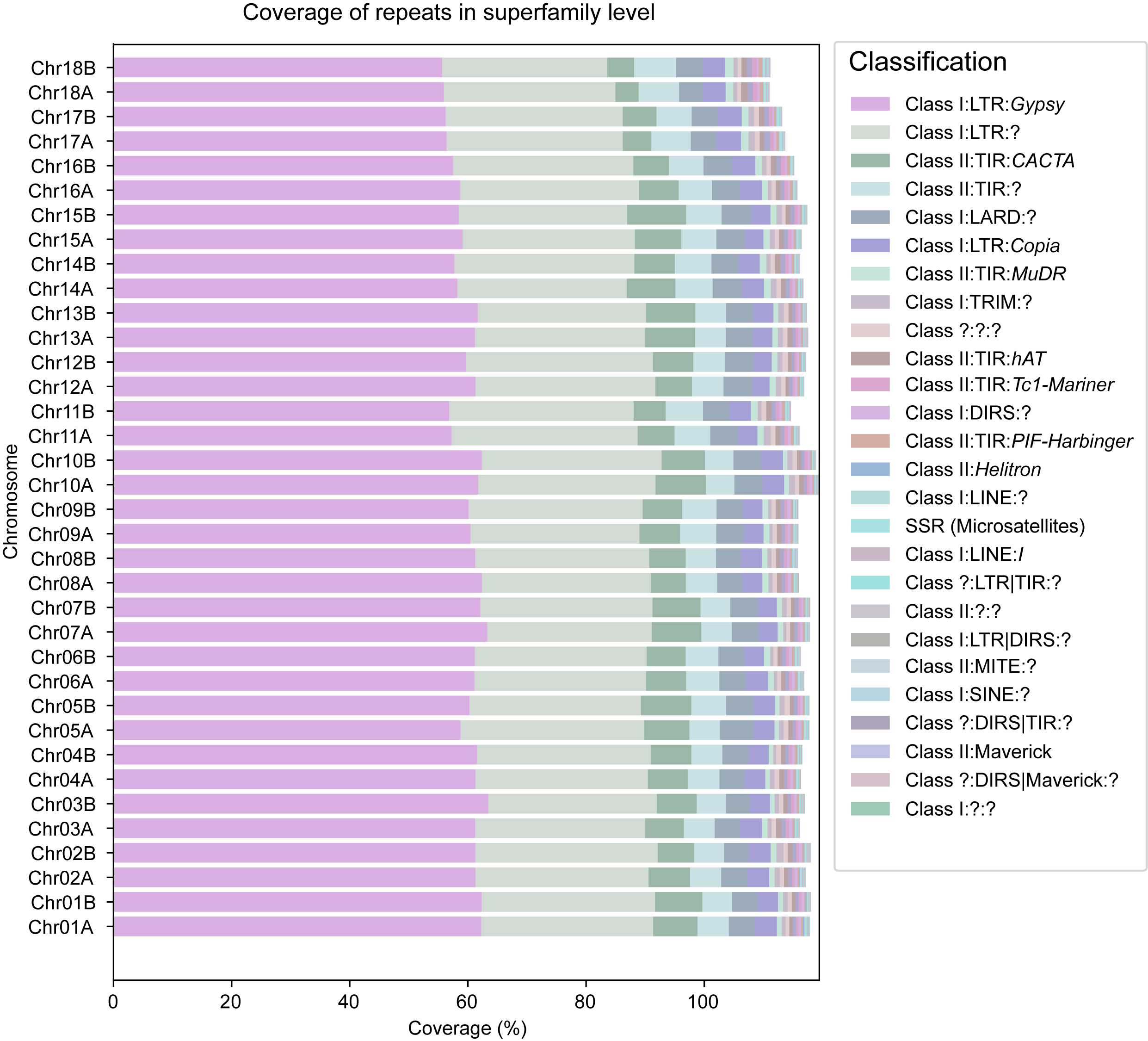

Supplement: jkaf255_Supplementary_Data [file jkaf255_supplementary_data.zip › Supplemental_Figure_4_G3-2025-406293.tif]

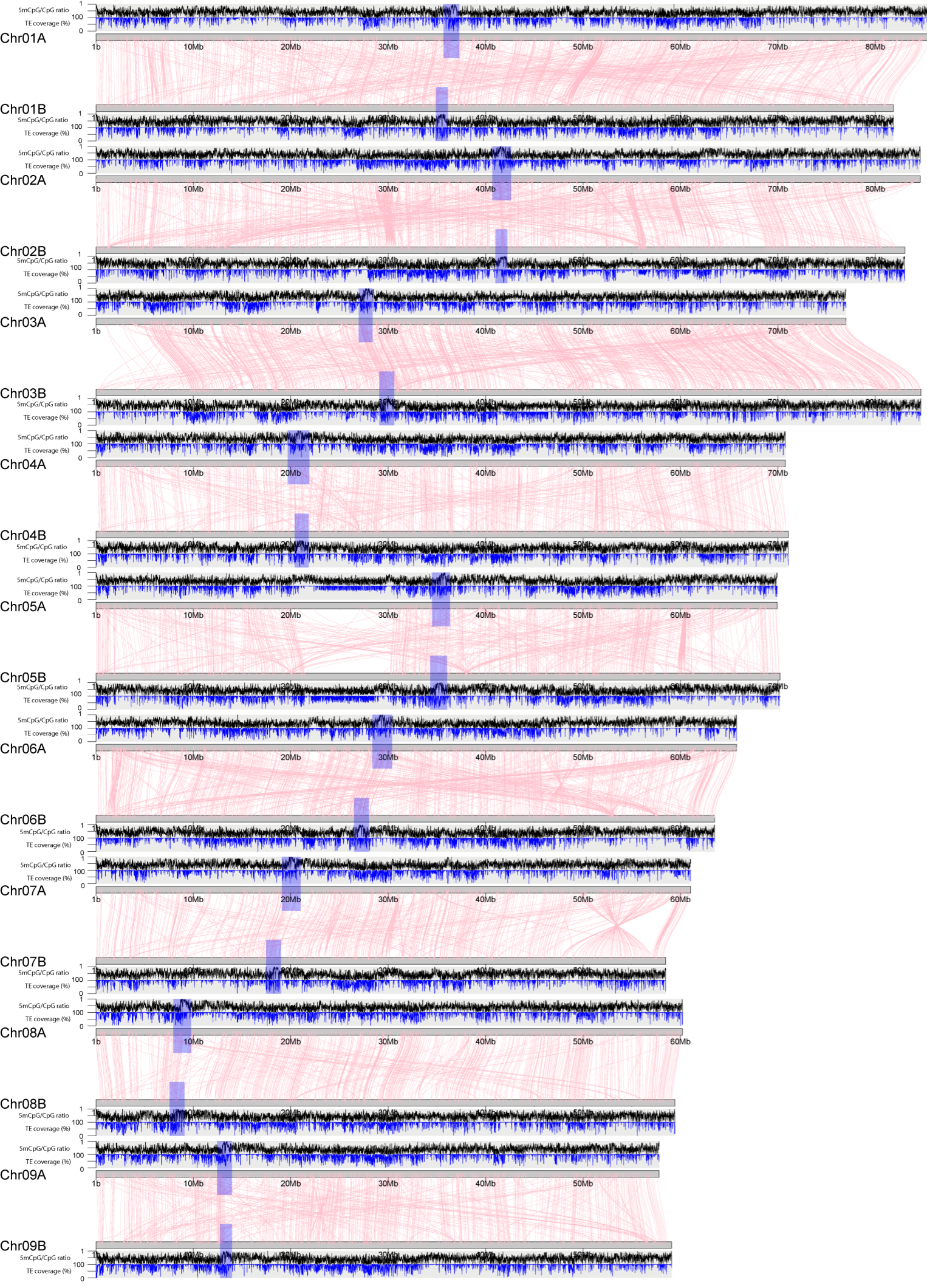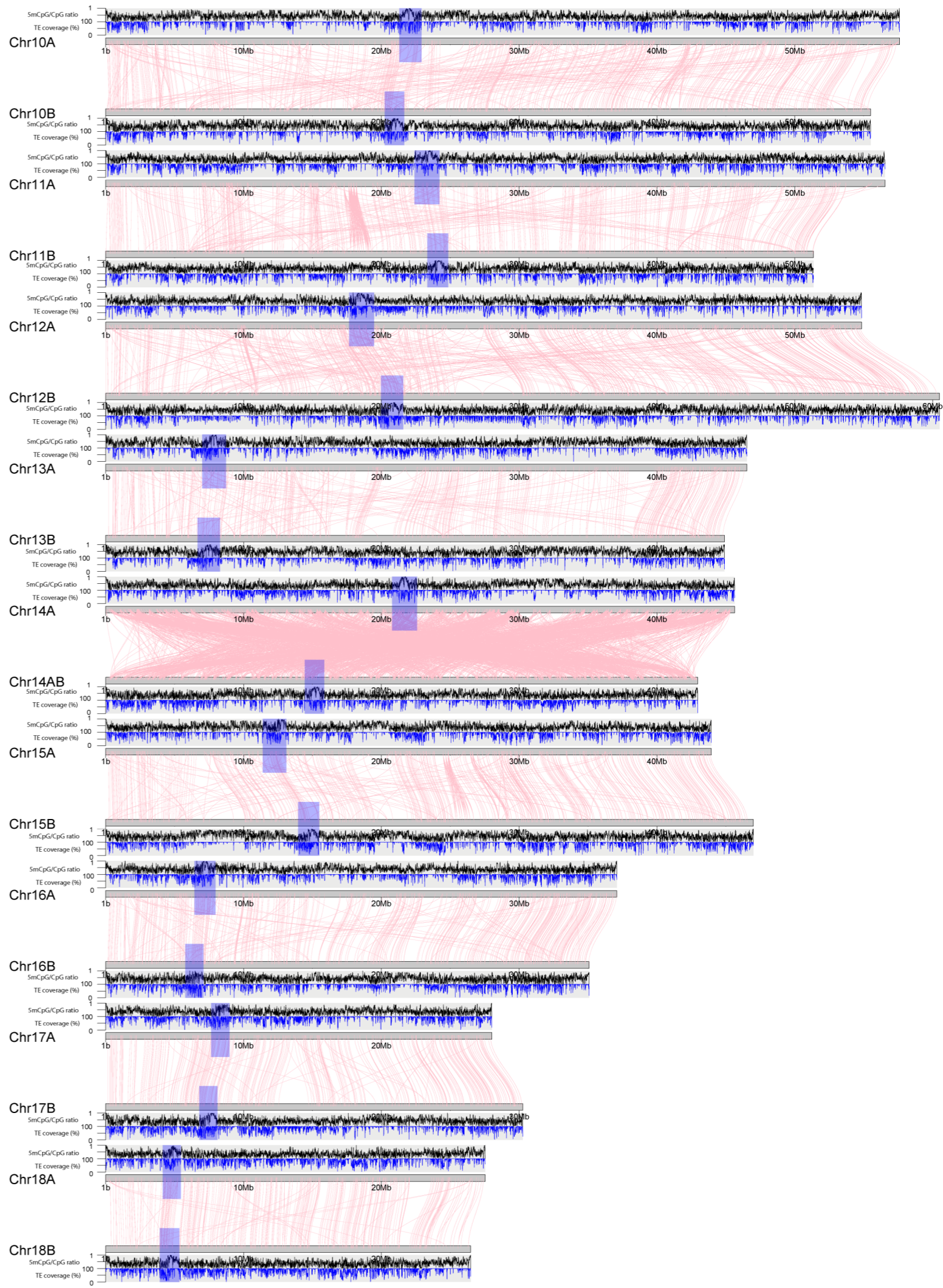

Supplement: jkaf255_Supplementary_Data [file jkaf255_supplementary_data.zip › Supplemental_Figure_5_G3-2025-406293.pdf]

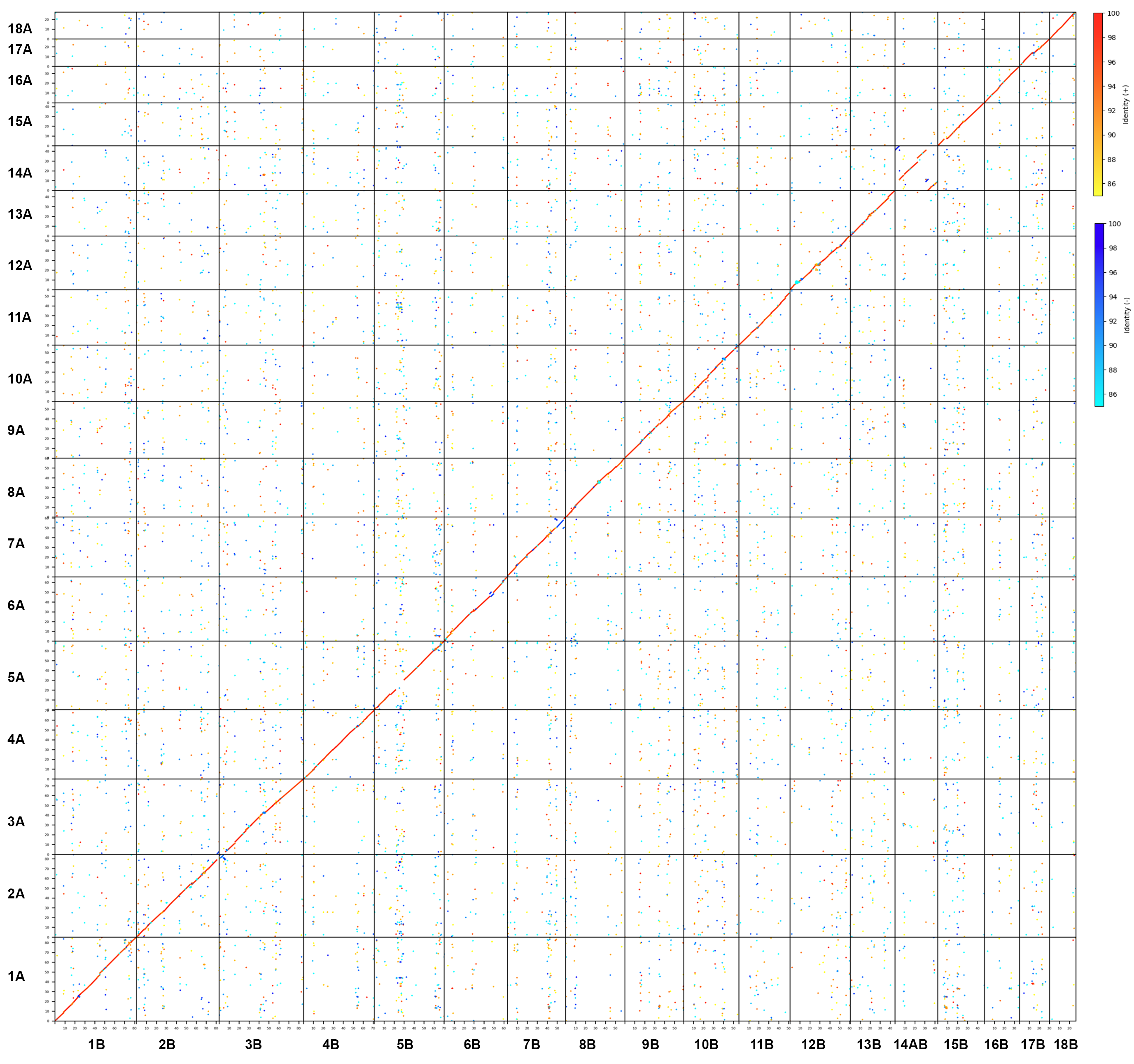

Supplement: jkaf255_Supplementary_Data [file jkaf255_supplementary_data.zip › Supplemental_Figure_6_G3-2025-406293.tif]

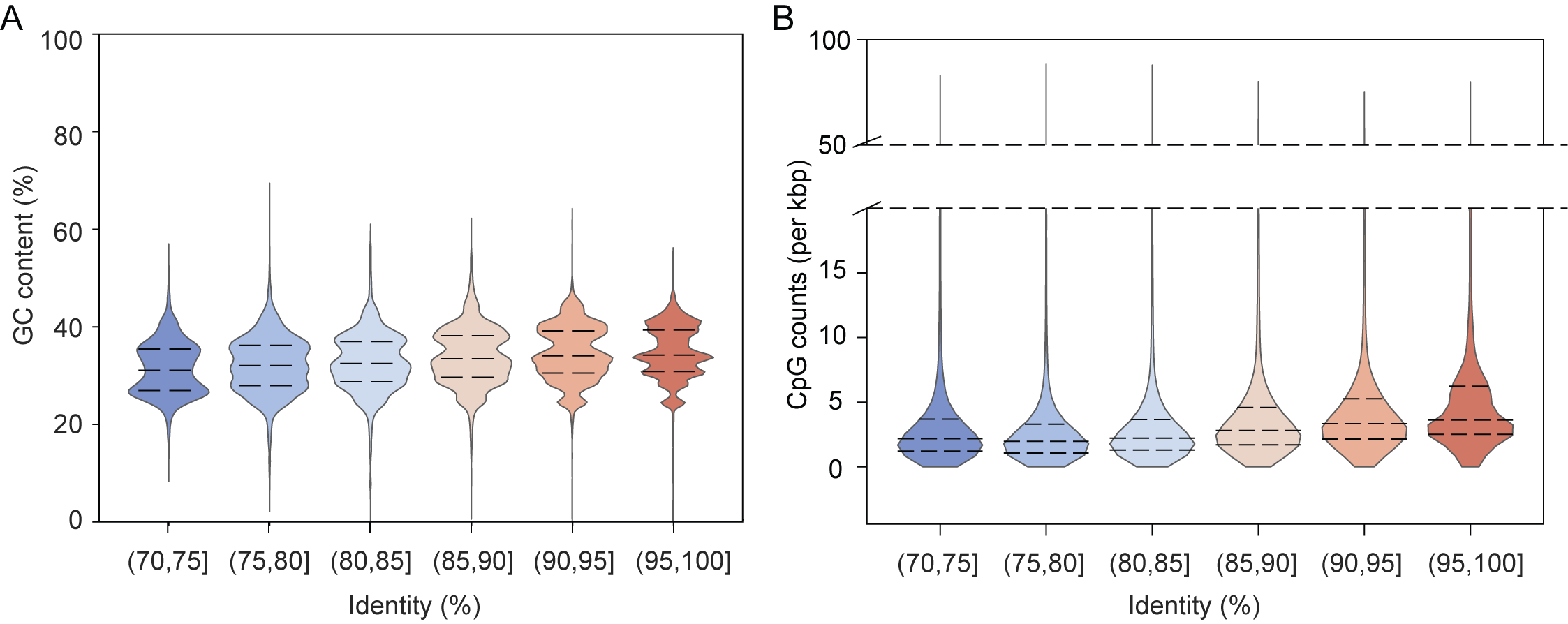

Supplement: jkaf255_Supplementary_Data [file jkaf255_supplementary_data.zip › Supplemental_Figure_7_G3-2025-406293.tif]

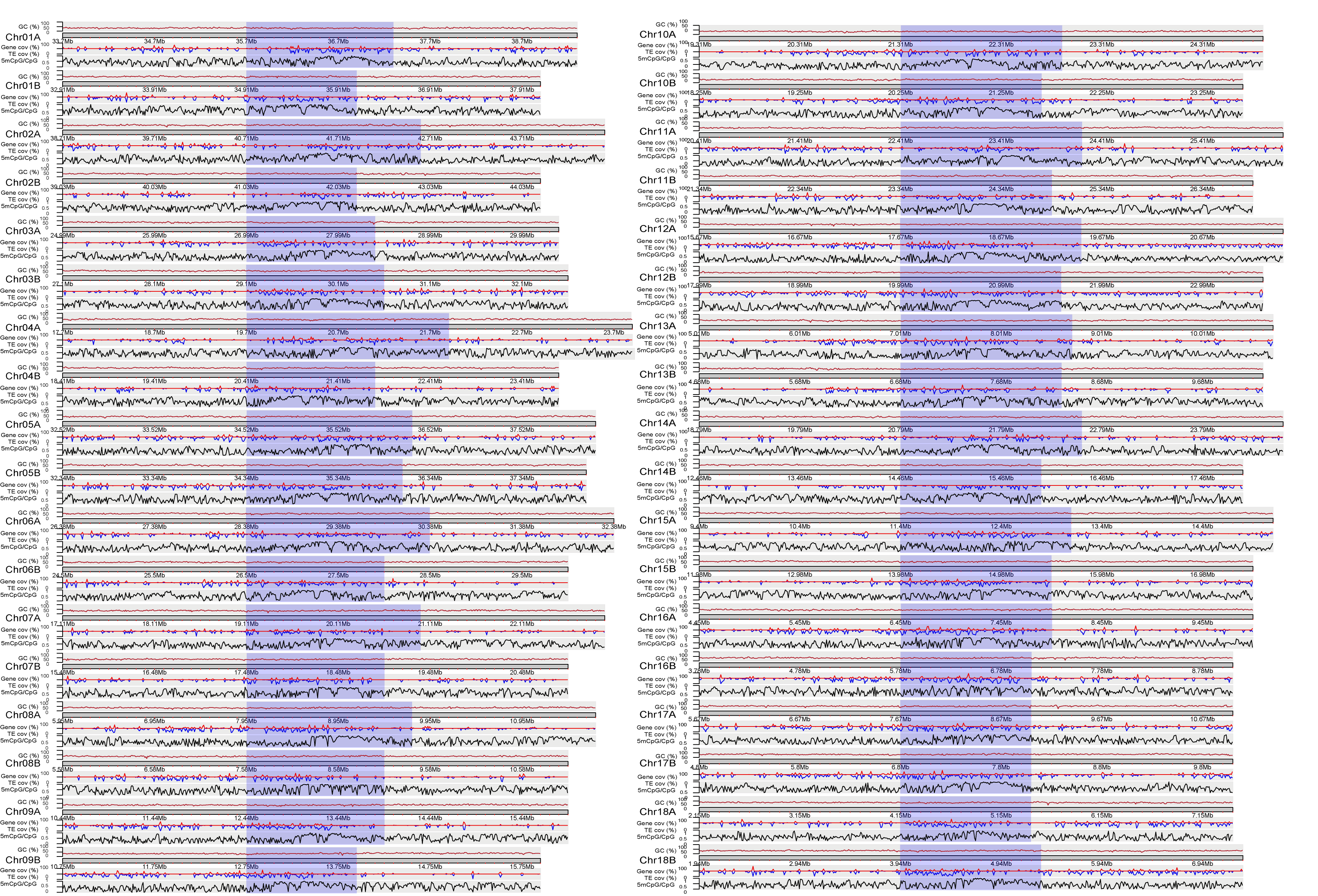

Supplement: jkaf255_Supplementary_Data [file jkaf255_supplementary_data.zip › Supplemental_Figure_8_G3-2025-406293.tif]

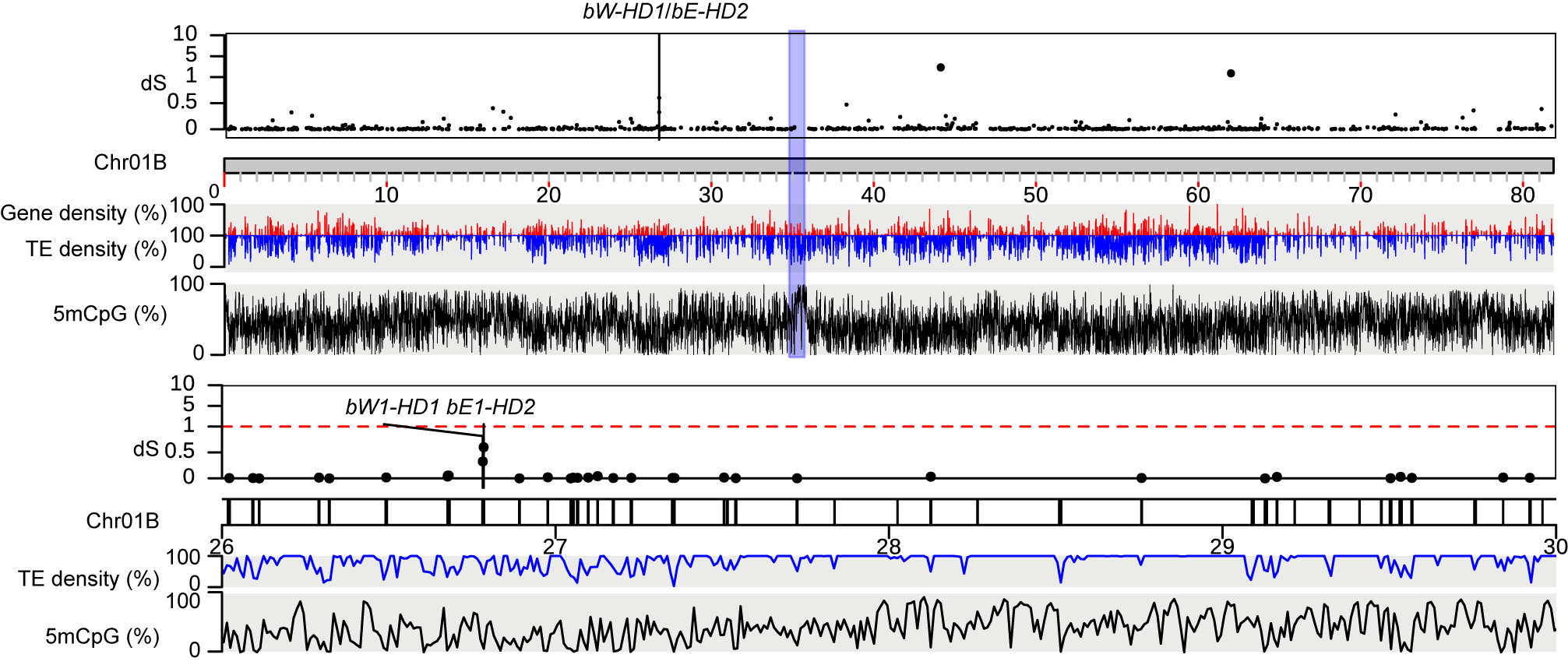

Supplement: jkaf255_Supplementary_Data [file jkaf255_supplementary_data.zip › Supplemental_Figure_9_G3-2025-406293.tif]
